# Integration of Moderation and Mediation in a Latent Variable Framework: A Comparison of Estimation Approaches for the Second-Stage Moderated Mediation Model

**DOI:** 10.3389/fpsyg.2020.02167

**Published:** 2020-09-10

**Authors:** Qingqing Feng, Qiongya Song, Lijin Zhang, Shufang Zheng, Junhao Pan

**Affiliations:** Department of Psychology, Sun Yat-sen University, Guangzhou, China

**Keywords:** latent moderated structural equations, product indicator, path analysis, moderated mediation model, approach comparison

## Abstract

An increasing number of studies have focused on models that integrate moderation and mediation. Four approaches can be used to test integrated mediation and moderation models: path analysis (PA), product indicator analysis (PI, constrained approach and unconstrained approach), and latent moderated structural equations (LMS). To the best of our knowledge, few studies have compared the performances of PA, PI, and LMS in evaluating integrated mediation and moderation models. As a result, it is difficult for applied researchers to choose an appropriate method in their data analysis. This study investigates the performance of different approaches in analyzing the models, using the second-stage moderated mediation model as a representative model to be evaluated. Four approaches with bootstrapped standard errors are compared under different conditions. Moreover, LMS with robust standard errors and Bayesian estimation of LMS and PA were also considered. Results indicated that LMS with robust standard errors is the superior evaluation method in all study settings. And PA estimates could be severely underestimated as they ignore measurement errors. Furthermore, it is found that the constrained PI and unconstrained PI only provide acceptable estimates when the multivariate normal distribution assumption is satisfied. The practical guidelines were also provided to illustrate the implementation of LMS. This study could help to extend the application of LMS in psychology and social science research.

## Introduction

Within education and psychology research, mediation and moderation effects are usually applied to gain a better understanding of the relationships between the predictors and outcomes. Mediation models can help in understanding the mechanisms of observed phenomena. In the mediated mechanism, an *indirect effect* is an effect of an independent variable on a dependent variable via a mediator variable; a *direct effect* is an effect of an independent variable on a dependent variable without a mediator variable (Baron and Kenny, 1986). Moderation models demonstrate that the effects of predictors on outcomes are dependent on the moderators (James and Brett, 1984; Baron and Kenny, 1986). The integrated model of mediation and moderation incorporates the properties of mediation and moderation simultaneously in a single study to explain the complexity of real world (e.g., Liao et al., 2010; Teper et al., 2013).

There are two main types of integrated models for mediation and moderation, as delineated by statisticians: moderated mediation and mediated moderation (Baron and Kenny, 1986; Edwards and Lambert, 2007). The differentiation between these two main types depends on the importance of the moderated and mediated effects within an experiment (Baron and Kenny, 1986; Edwards and Lambert, 2007). In recent years, there have been many practical studies applying an integrated moderation and mediation approach. For example, this study identified 203 original articles with the keywords “moderated mediation” and 44 articles with the keywords “mediated moderation” published in the PsychArticle database between January 2000 and January 2020. Given that the majority of articles applied the moderated mediation model, this became the focus of this study. This model has a latent interaction effect between moderator and mediator variables, thus qualifying it as a representation of the integrated model. The method for testing a mediated effect in the integrated model is similar to that in the simple mediation model, which is easy for researchers to master. However, the moderated effect is much more difficult to calculate in this complex model than in the simple moderated model. Therefore, this study concentrates on the estimation of the path coefficient of the interaction effect under the second-stage moderated mediation model.

Throughout the study, variables correspond to the following basic equations:

(1) $$\begin{array}{l} \text{M}=\text{a}X+\delta_{M}\\ \text{Y}=cX+b_{1}M+b_{2}Z+b_{3}MZ+\delta_{Y} \end{array}$$

This study uses X to represent the independent variable; Y to represent the dependent variable; M to represent the mediator, which mediates the relationship between X and Y; and Z to represent the moderator, which moderates the process of M predicting Y. According to the equations, the path coefficient from X to M is *a* and the path coefficient from X to Y is *c*. These two effects are classified as direct effects. The path coefficient from M to Y is *b*_1_, and from Z to Y it is *b*_2_. The parameter of the relationship from the interaction effect MZ to the dependent variable Y is *b*_3_.

**Figure 1:**
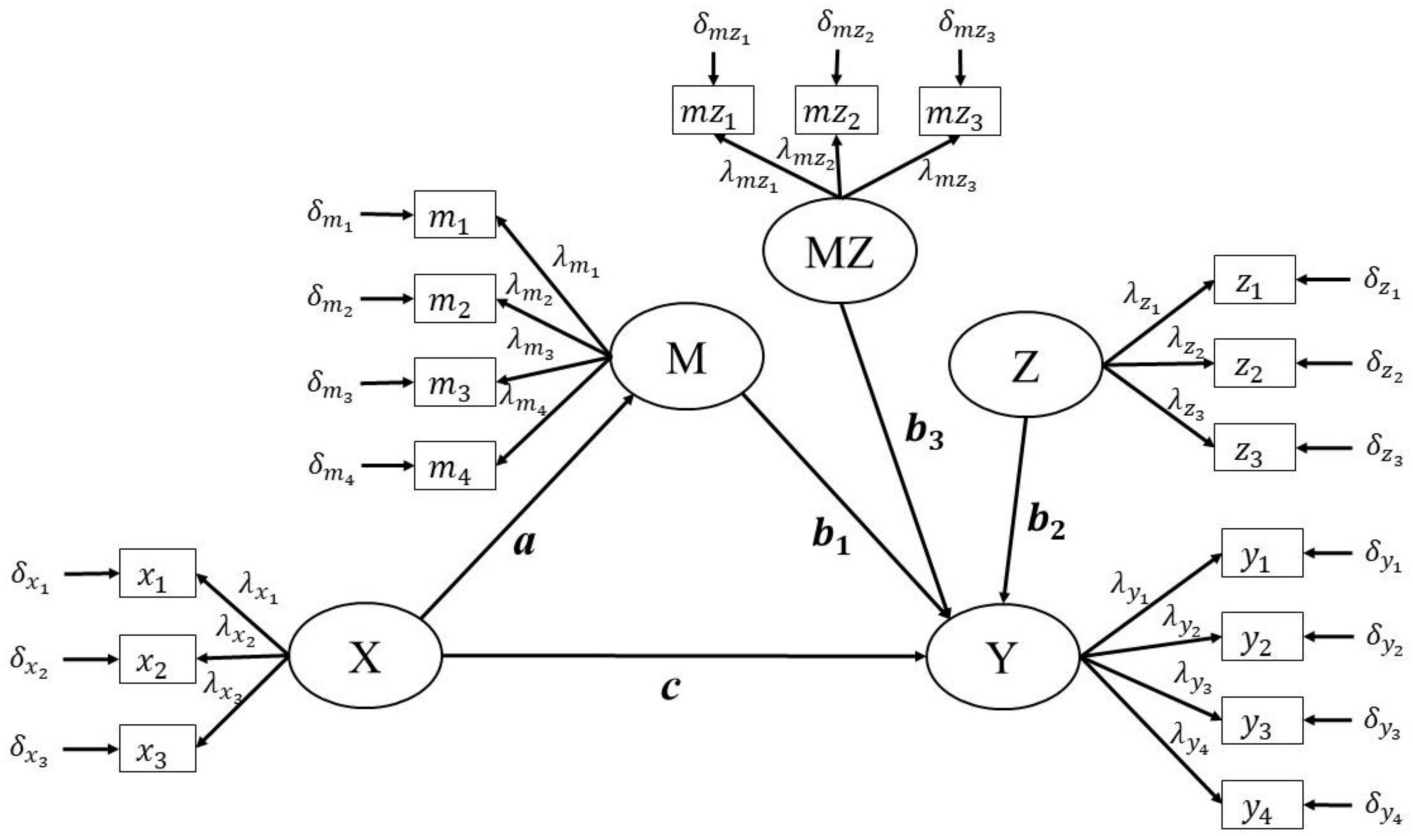
The population model of the Product Indicator approach's paring strategy of indicators.

Many researchers have discussed and applied the integrated moderation and mediation model in their studies, based on the manifest variable framework (e.g., Edwards and Lambert, 2007; Hayes, 2013; Fang et al., 2014). However, there has been less discussion and application of the approaches within the latent variable framework (Cheung and Lau, 2017). Within the latent variable framework, a measurement model is built to relate each latent variable to its corresponding indicators (Figure 1). We assume that the independent variable X and the moderator variable Z each have three indicators, and that the dependent variable Y and the mediator variable M each have four indicators. This assumption is only required to approximate an actual study. In reality, a study may not have three indicators with a just-identified model for every latent variable. Furthermore, different indicators for the variables M and Z, which would be used to form an interaction MZ, could increase the complexity in some approaches when forming the latent interaction term MZ. Taking the latent variable X as an example, its measurement model can be represented as:

(2) $$\begin{array}{l} \begin{array}{c} \left(\begin{array}{c} x_{1}\\ x_{2}\\ x_{3} \end{array}\right)=\left(\begin{array}{c} \tau_{1}\\ \tau_{2}\\ \tau_{3} \end{array}\right)+\left(\begin{array}{c} \lambda_{1}\\ \lambda_{2}\\ \lambda_{3} \end{array}\right)X+\left(\begin{array}{c} \delta_{x_{1}}\\ \delta_{x_{2}}\\ \delta_{x_{3}} \end{array}\right) \end{array} \end{array}$$

where *x*_1_ to *x*_3_ are the indicators of latent variable *X*, τs are the intercepts, and λs are the factor loadings. The error variance is represented as Var(δ_*x*_*s*__) and the variance of latent variable X is Var(X). Similarly, the measurement models for Y, M, and Z can be defined in the same way.

It is inherently difficult to estimate the interaction effect in the integrated moderation and mediation model due to the complicated non-linear constraints in the latent framework (Jöreskog and Yang, 1996). Although many approaches have been developed and shown to work quite well in estimating the latent interaction effect (e.g., Klein and Moosbrugger, 2000; Algina and Moulder, 2001; Wall and Amemiya, 2001), a complete comparison of the approaches to clarify the optimal choice has not been conducted. This study addresses this research gap. Two simulation studies with different conditions are developed to evaluate the various methods for estimating the second-stage moderated mediation model. The approaches examined here were path analysis (PA), constrained product indicator analysis (CPI), unconstrained product indicator analysis (UPI), and latent moderated structural modeling (LMS).

## Methodology and Design

### Path Analysis (PA)

Many previous researchers have provided procedures to use traditional regression methods to analyze moderated mediation (e.g., Muller et al., 2005; Edwards and Lambert, 2007; Preacher et al., 2007; Hayes, 2013). In contrast to traditional regression analysis, PA can simultaneously estimate all effects and integrate them into a single complete model framework. Based on this framework, PA uses the means of indictors to represent the value of the corresponding latent variables. For example:

(3) $$\begin{array}{l} \begin{array}{c} \text{X}=\frac{x_{1}+x_{2}+x_{3}}{3} \end{array} \end{array}$$

Thus, the interaction term could be computed as:

(4) $$\begin{array}{l} \begin{array}{c} \text{MZ}=\frac{m_{1}+m_{2}+m_{3}+m_{4}}{4}\times \frac{z_{1}+z_{2}+z_{3}}{3} \end{array} \end{array}$$

PA has been employed extensively in practical research because it is easy to grasp and convenient to apply (Cheung and Lau, 2017). However, the use of PA has also been opposed due to its lack of consideration of measurement errors. Cheung and Lau (2008) have previously found that simple regression analysis for a mediation model underestimates the coefficients and the extent of underestimation increases as measurement errors increase. Statisticians have recommended methods within a latent framework to reduce such inaccuracy resulting from measurement errors (e.g., Marsh et al., 2004; Klein and Muthén, 2007; Wen et al., 2010). Considering the complicated latent interaction effect, measurement errors may lead to more bias in the integration model (Ree and Carretta, 2006; Cheung and Lau, 2008; Antonakis et al., 2010; Ledgerwood and Shrout, 2011).

### Product Indicator Analysis (PI)

Within the latent variable framework, Kenny and Judd (1984) proposed the idea of measuring a latent interaction effect by multiplying the indicators of two latent variables. On the basis of this concept, many statisticians have proposed and developed various approaches to estimate interaction effects using PI (e.g., Jöreskog and Yang, 1996; Klein and Moosbrugger, 2000; and Wall and Amemiya, 2001, 2003; Marsh et al., 2004). PI has proven to be more accurate than the traditional regression method and PA, as it considers the measurement errors of the product indicators (Jöreskog and Yang, 1996).

However, in practical research, PI has not been used as often as PA. There are two main reasons that the application of PI has been limited. One is that PI is quite difficult to grasp and carry out. The use of PI requires many extra constraints to identify the model. Although previous researchers have already developed the UPI approach (Marsh et al., 2004), many applied researchers still find it difficult to implement. The other reason that the application of PI has been limited is that it is difficult to choose and execute the most appropriate method to form the product indicators for the latent interaction effect (Marsh et al., 2004).

In this study, it is assumed that the three indicators of M and first three of the four indicators of Z were multiplied to form the indicators for the latent interaction factor, as detailed in the following equation:

(5) $$\begin{array}{l} \begin{array}{c} \left(\begin{array}{c} mz_{1}\\ mz_{2}\\ mz_{3} \end{array}\right)=\left(\begin{array}{c} \tau_{mz_{1}}\\ \tau_{mz_{2}}\\ \tau_{mz_{3}} \end{array}\right)+\left(\begin{array}{c} \lambda_{mz_{1}}\\ \lambda_{mz_{2}}\\ \lambda_{mz_{3}} \end{array}\right)MZ+\left(\begin{array}{c} \delta_{mz_{1}}\\ \delta_{mz_{2}}\\ \delta_{mz_{3}} \end{array}\right) \end{array} \end{array}$$

Here, τ_*m*_*z*__*s*__s represent the intercepts of the product indicators, λ_*m*_*z*__*s*__s represent the factor loadings of the latent interaction, *MZ* represents the latent interaction factor, and Var(δ_*m*_*z*__s__) represents the error variances.

#### Constrained Product Indicator Analysis (CPI)

Jöreskog and Yang (1996) introduced a strong constrained model, called the constrained product indicator (CPI) approach, of the latent interaction effect in order to identify the model. This approach assumes that indicators are centralized and multivariate normally distributed (Algina and Moulder, 2001). For example, the first (product) indicator for the latent interaction *MZ* could be represented as:

(6) $$\begin{array}{l} mz_{1}=\lambda_{mz_{1}}MZ+\delta_{mz_{1}}\\ \;=\left(\lambda_{m_{1}}M+\delta_{m_{1}}\right)\left(\lambda_{z_{1}}Z+\delta_{z_{1}}\right)\\ \;=\lambda_{m_{1}}\lambda_{z_{1}}MZ+\left(\lambda_{m_{1}}M\delta_{z_{1}}+\lambda_{z_{1}}Z\delta_{m_{1}}+\delta_{m_{1}}\delta_{z_{1}}\right) \end{array}$$

where the factor loadings and error variances of the indicator *mz*_1_ could be considered as functions of the factor loadings and error variances of the indicators of M and Z. The constraints of CPI consist of the variance and mean of the latent interaction of MZ, as well as the factor loadings and error variances of the indicators for MZ (Moulder and Algina, 2002; Marsh et al., 2004).

*The constraint of the factor loading of indicator* *mz*_1_ *for MZ* is:

(7) $$\begin{array}{l} \begin{array}{c} \lambda_{mz_{1}}=\lambda_{m_{1}}\times \lambda_{z_{1}} \end{array} \end{array}$$

Based on Equation 6, the error variances of the product indicators are not freely estimated.

*The constraint of the error variances of indicators* *mz*_1_ *for MZ* could be expressed as:

(8) $$\begin{array}{l} Var\left(\delta_{mz_{1}}\right)=Var\left(\lambda_{m_{1}}M\delta_{z_{1}}+\lambda_{z_{1}}Z\delta_{m_{1}}+\delta_{z_{1}}\delta_{m_{1}}\right)\\ \;=Var\left(\lambda_{m_{1}}M\delta_{z_{1}}\right)+Var\left(\lambda_{z_{1}}Z\delta_{m_{1}}\right)+Var\left(\delta_{z_{1}}\delta_{m_{1}}\right)\\ \;=\lambda_{m_{1}}^{2}Var\left(M\right)Var\left(\delta_{z_{1}}\right)+\lambda_{z_{1}}^{2}Var\left(Z\right)Var\left(\delta_{m_{1}}\right)\\ \;+Var\left(\delta_{z_{1}}\right)Var\left(\delta_{m_{1}}\right) \end{array}$$

*The constraint of the variance of MZ* is as follows:

(9) $$\begin{array}{l} Var\left(MZ\right)=Var\left(M\right)\;Var\left(Z\right)+{\left[Cov(M,Z)\right]}^{2}\\ \;=Var\left(aX+\delta_{M}\right)\;Var\left(Z\right)+{\left[Cov\left(aX+\delta_{M}\right),Z\right]}^{2}\\ \;=\left[Var\left(aX\right)+Var\left(\delta_{M}\right)\right]\;Var\left(Z\right)+[Cov\left(aX,Z\right)\\ \;{+Cov\left(\delta_{M},Z\right)]}^{2}\\ \;=\left[a^{2}Var\left(X\right)+Var\left(\delta_{M}\right)\right]\;Var\left(Z\right)\\ \;+{\left[aCov\left(X,Z\right)\right]}^{2} \end{array}$$

*The constraint of the mean of latent variable MZ* could also be expressed as:

(10) $$\begin{array}{l} E\left(MZ\right)=Cov\left(M,Z\right)+E\left(M\right)E\left(Z\right)\\ \;=Cov\left[\left(aX+\delta_{M}\right),Z\right]\\ \;=aCov\left(X,Z\right) \end{array}$$

However, Jöreskog and Yang's (1996) strong constrained model was too complex, given the increasing number of indicators. Therefore, it is difficult for most researchers to apply the model in real studies. Another limitation of the CPI approach is the assumption of the multivariate-normal distributed indicators. Although every indicator of the variable is normally distributed, the indicator for the latent interaction based on multiplication may not obey the normal distribution (Aroian, 1947; Moosbrugger et al., 1997). It is not clear how the non-normally distributed indicators of interaction would bias the estimation of the CPI approach.

#### Unconstrained Product Indicator Analysis (UPI)

Statisticians have considered various ways to relax the constraints and simplify the PI model, such as the partial constrained approach (Wall and Amemiya, 2001), the unconstrained approach (Marsh et al., 2004), and the expanded unconstrained approach (Kelava and Brandt, 2009). The unconstrained approach proposed by Marsh et al. (2004, 2013), which only requires the constraints of the latent mean (Equation 10), is commonly utilized. This is what is referred to as the UPI approach in this paper.

An advantage of the UPI approach is that it only requires the mean of the latent interaction to be fixed, which makes the approach easy to apply, even for complicated models. Additionally, simulation studies have identified that the UPI approach provides a more precise estimation than the CPI approach when data violate the multivariate normality assumption. However, compared to the Algina and Moulder (2001) revised strict constrained model, the UPI approach showed less power in the estimation, even under the multivariate normality assumption (Marsh et al., 2004).

In sum, there are four types of constraints involved in the PI approach: (1) the constraints of the factor loadings of the product indicators; (2) the constraints of the error variances of the product indicators; (3) the constraint of the variance of the latent interaction factor; and (4) the constraint of the mean of the latent interaction factor. Jöreskog and Yang (1996) CPI approach requires all four constraints, whereas Marsh et al. (2004) UPI approach requires a minimum of only one constraint, that is, the mean of the latent interaction should to be fixed. This study aims to compare these two approaches to evaluate the performance for the constrained form of the PI approach.

Another limitation of the PI approach is the difficulty researchers face in choosing among multiple indicators to construct the product indicators for the latent interaction factor (Marsh et al., 2004). Different strategies to construct the product indicators in the same model may perform differently (e.g., Marsh et al., 2004; Saris et al., 2007; Aytürk et al., 2019). Researchers need to choose suitable strategies to get a reasonable result, which makes the application of the PI approach a challenge.

#### Strategies to Construct Product Indicators

There are many strategies to construct product indicators. The most widely applied strategies are Matching and Parceling (e.g., Marsh et al., 2004; Aytürk et al., 2019).

##### Matching

Indicators are paired according to factor loadings. This means that the indicators with larger factor loadings for M need to be multiplied with the indicators with larger factor loadings for Z. In this study, we could produce three product indicators for the latent interaction factor.

##### Parceling

A parcel means an aggregation (e.g., average) of the indicators for the latent factors. The parceling strategy could help reduce the number of indicators by using the parcels to replace the original indicators. However, there are also many strategies to form the parcels, such as putting the indicators of the highest and the lowest factor loadings in one parcel or randomly setting the indicators.

Since the comparison between matching and parceling is not the focus of the current study, applied researchers are suggested to refer to previous studies for guidelines. Aytürk et al. (2019) pointed out that parceling has advantages of considering all the indicators while matching ignoring the remaining indicators which are not used for generating interaction terms. Since the number of indicators per factor is small in the current study, a matching strategy with multiple indicators, using indicators for M and Z with the highest factor loading, will be used (Marsh et al., 2004). The matching approach would be repeated until all indicators of one variable are matched (Figure 1).

### Latent Moderated Structural Equation Modeling (LMS)

Klein and Moosbrugger (2000) proposed the LMS equation modeling to address the estimation of the latent interaction model. The LMS approach constructs the latent interaction effect as a conditioned linear effect on another variable. It simply utilizes the original data to estimate the interaction, without introducing product indicators (Kelava et al., 2011; Aytürk et al., 2019).

LMS not only takes measurement errors into account, but also solves the two issues affecting the PI approach. LMS could prevent researcher frustration in terms of having to choose which strategy to use in constructing the product indicators. Furthermore, LMS does not require any extra non-linear constraints to identify the model and does not need the interaction indicators to obey the strict multivariate normally distributed assumption (Klein and Moosbrugger, 2000; Kelava et al., 2011). Many previous simulation studies have already proven that LMS can provide precise parameter and standard error estimates with smaller biases (Schermelleh-Engel et al., 1998; Klein and Moosbrugger, 2000). However, most mainstream analysis software cannot apply LMS with real study data. Issues are experienced in forming the conclusion for LMS, even with M*plus*. For example, M*plus* cannot export model fit indices that can be used to directly evaluate the model fitting of moderated mediation models (e.g., comparative fit index, CFI). These issues decrease the attractiveness of LMS for applied researchers.

### Bayesian Estimation

Apart from the traditional maximum likelihood (ML) estimation, Bayesian estimation has also been demonstrated efficient in structural equation modeling and has recently become increasingly popular (van de Schoot et al., 2017). Compared with the ML estimator, Bayesian estimation is based on a sampling method rather than on asymptotic theory (large-sample theory), and thus performs better with small sample size (Muthén and Asparouhov, 2012). Moreover, without a strict assumption of normal distribution, the Bayesian method is also expected to have better performance with non-normally distributed data. In this research, to simply compare the Bayesian estimation with the ML estimator, we used both of them in LMS and PA since the CPI and UPI models cannot be conducted using Bayesian method in M*plus* 8.4^1^. Default settings of M*plus* with non-informative priors were used except for the minimum number of iterations (which was set at 10,000) in Bayesian estimation. The default non-informative prior for the intercepts of items, factor loadings and path coefficients are *N*(0, infinity). For the variance-covariance matrix of latent variables, the default prior is inverse-Wishart(0, -*p*-1) where *p* is the dimension of the multivariate block of latent variables (Muthén and Muthén, 1998-2017). We obtained the point estimates using the median of posterior distribution and calculated the 95% credible interval based on the percentile method.

### Research Design

For the present research, two simulation studies with different conditions were conducted. In the first study, the data were generated to follow a normal distribution and have high/low reliability. Additionally, our definition called for no correlation between the moderator variable, Z, and the independent variable, X. In the second study, non-normally distributed data were taken into consideration. This study explored the tendency of estimates to be biased for non-normal distributions with different skews and kurtoses. The correlation of the independent variable, X, and the moderator variable, Z, was also considered in the second study.

The four approaches: PA, CPI, UPI, and LMS in these studies were compared. Since the bootstrap method is often required in mediation analysis, we obtained the bootstrapped standard errors and confidence intervals in four approaches using 100 bootstrap draws^2^, which is suggested as the least number of draws in the bootstrap method (Rutter, 2000). Since the bootstrap method is not available for the MLR estimator (Maximum Likelihood estimation with Robust standard errors), which is the default estimation method in LMS with M*plus*, we also calculated the robust standard errors in LMS and compared this with the bootstrap method. As mentioned above, the LMS and PA approaches with Bayesian estimation were also taken into consideration.

## Study 1

### Population Model

As per the research design of Marsh et al. (2004), data are generated with a normal distribution. The settings for this are as follows: The population model in simulation studies is the same as that in Figure 1 except MZ does not have its indicator.

The factor loadings of the independent variable, X (λ_*x*_1__, λ_*x*_2__, and λ_*x*_3__), are 1.0, 0.65, and 0.72, respectively. The error variance of the indicators (Var(δ_*x*_*s*__)) are all set to 0.36/1.5. The intercepts (τ_*x*_*s*__) are 0.5 and the variance of X (Var(X)) is 1.0.

For the mediator variable M, the factor loadings of the four indicators (λ_*m*_1__, λ_*m*_2__, λ_*m*_3__, and λ_*m*_4__) are 1.0, 0.81, 0.53, and 0.66, respectively. The error variances (Var(δ_*m*_*s*__)) are all 0.36/1.5. The intercepts (τ_*m*_*s*__) are set to 0.5 and the residual variance of M (Var(δ_*M*_)) is 0.36.

The indicators of the moderator variable, Z, have factor loadings (λ_*z*_1__, λ_*z*_2__, and λ_*z*_3__) of 1.0, 0.83, and 0.79, respectively. The error variances of the indicators (Var(δ_*z*_*s*__)) are all 0.36/1.5. The intercepts (τ_*z*_*s*__) are set to 0.5 and the variance of Z (Var(Z)) is 1.0.

The dependent variable, Y, has four indicators with factor loadings (λ_*y*_1__, λ_*y*_2__, λ_*y*_3__, and λ_*y*_4__) set as 1.0, 0.68, 0.75, and 0.83, respectively. The error variances (Var(δ_*y*_*s*__)) are all 0.36. The intercepts of the four indicators (τ_*y*_*s*__) are set to 0.5, and the residual variance of Y (*Var*(δ_Y_)) is 0.36/1.5.

Finally, the path coefficients in Equation 1 are set as follows: *a* = 0.75, *c* = 0.3, *b*_1_ = 0.56, and *b*_2_ = 0.48.

### Design

This study has a 4^*^3^*^2 design with a manipulation of the sample sizes, the effect of the interaction term, and the reliability of latent variable. The four levels of sample sizes are: (a) *N* = 100, (b) *N* = 200, (c) *N* = 500, and (d) *N* = 1,000. These sample sizes are typical in psychological research. *N* = 100 is considered the minimum sample size for SEM, *N* = 200 is a common, medium sample size (Boomsma, 1982), and a sample of 500 is considered large enough to provide unbiased estimates for most applied research (Kyriazos, 2018). Moreover, Cham et al. (2012) suggested that the sample size for latent moderation modeling should be relatively large, so the sample size of 1,000 was also considered in the current study.

There are also three levels of path coefficients of *b*_3_: (a) *b*_3_ = 0, (b) *b*_3_ = 0.2, and (c) *b*_3_ = 0.4. Among these settings, *b*_3_ = 0 is intended to test for the Type I error, and *b*_3_ = 0.2 and 0.4 are intended to test the power of the four approaches (Klein and Moosbrugger, 2000).

We set the error variances of the indicators to control for the reliability of the latent variable, and under high reliability conditions, the error variances of the indicators are all set to 0.36. The mean reliability of the four latent variables X, M, Z, and Y were 0.822 (SD = 0.013), 0.836 (SD = 0.012), 0.859 (SD = 0.010), and 0.896 (SD = 0.008) in the condition of *N* = 500 and *b*_3_ = 0.2. All variables are identified to have high reliability. Under low reliability conditions, the error variances (δs) were changed to 1.5 in accordance with previous research by Cheung and Lau (2017). The mean reliability of the latent variables, X, M, Z, and Y, were set as 0.542 (SD = 0.037), 0.569 (SD = 0.033), 0.601 (SD = 0.030), and 0.684 (SD = 0.024), respectively, in the same condition. The mean reliability of X, M, Z, and Y barely changed across the same (high or low) reliability conditions, demonstrating that the manipulation of reliability was successful.

This study aims to test the four approaches with the bootstrap method in the 24 conditions with the generated data. The simulation data are generated by the statistical program R (version 3.5.3). The 24 conditions each have 1,000 replications. The Monte Carlo experiments were conducted using M*plus* 8.4 (Muthén and Muthén, 1998-2017).

### Evaluation Measures

In the simulation study, the performance of the approaches is evaluated using five different measures: relative bias of parameter estimates, standard error (SE) ratio, coverage rate, power and type I error, and completion rate. These are all common assessment metrics for the accuracy of parameter estimates.

#### Relative Bias of Parameter Estimates

The relative bias measure is the relative bias between the estimated value and the true value of a parameter. The equation for relative bias, represented by RB, is:

(11) $$\begin{array}{l} \begin{array}{c} RB=\frac{\hat{\theta }-\theta }{\theta } \end{array} \end{array}$$

where $\hat{\theta }$ is the estimated value and θ is the parameter's true value. |*RB*| ≤ 10 % is a threshold typically accepted by researchers (Flora and Curran, 2004).

#### Standard Error (SE) Ratio

The SE ratio is the ratio between the mean of the standard error of $\hat{\theta }$ in 1,000 replications and the standard deviation of $\hat{\theta }$ across 1,000 replications, which is defined as:

(12) $$\begin{array}{l} \begin{array}{c} SE\;ratio=\frac{\bar{SE\left(\hat{\theta }\right)}}{SD\left(\hat{\theta }\right)} \end{array} \end{array}$$

A ratio between 0.9 and 1.1 is usually accepted as representing a relatively precise estimation (Hoogland and Boomsma, 1998).

#### Coverage Rate

The coverage rate is the probability that the estimate values will fall in the 95% (bootstrapped) confidence interval/Bayesian credible interval. Usually, the coverage rate needs to be over 90% to ensure the likelihood that the parameter estimation results are included (Collins et al., 2001). The coverage rate relates to the estimation's relative bias and its SE.

#### Power and Type I Error

Power is the probability of correctly rejecting the false null hypothesis; that is, when parameters are non-zero. On the contrary, if the null hypothesis is true, then the probability of the false rejection of the null hypothesis is the type I error when the true values of parameters are zero.

The acceptable level of power is above 80%. The Type I error rate should be within the 95% confidence interval of a binomial variable [0.0365, 0.0635] ($0.05\pm 1.96\times \sqrt{0.05\times \left(1-0.05\right)/number\;of\;replications}$; Cham et al., 2012).

#### Completion Rate

The completion rate is the proportion of replications with fully proper solutions in the 1,000 replications for each condition.

### Results

Marsh et al. (2004) found that the change of the interaction term does not heavily influence the estimation of the main effect. Results in the current study also demonstrated the same conclusion except for the PA method. Thus, to save space, we focus on the estimates of the path coefficient *b*_3_ and the moderated mediation index (*a* * *b*_3_, Hayes, 2015), and the estimates of indirect effects at different values of the moderator (*a* * (*b*_1_ + *b*_3_*Z), *when Z* = 0, 1 *SD*, − 1 *SD*) in the four analysis approaches. The comparison applies the five evaluation measures. The detailed results of the main effects are available in the Supplemental Materials. All the simulation materials including data generation, analysis, evaluation measurements and so on are also uploaded as the Supplemental Materials.

#### Relative Bias

As detailed in Tables 1–3, PA with bootstrap method / Bayesian estimation underestimated the parameters which are not zero (e.g., *b*_3_= 0.2 and 0.4, the average indirect effects), and the relative biases are unacceptable when reliability is low. The average estimates of indirect effects and moderated mediation index were also seriously biased using path analysis even when the reliability was high. This result aligns with previous researchers' conclusions about PA regarding parameter underestimation (Cheung and Lau, 2017).

**Table 1:** Results of Simulation Study 1 when *b*_3_ = 0.

***N***		**Estimator**	**High reliability**				**Low reliability**			
			**100**	**200**	**500**	**1,000**	**100**	**200**	**500**	**1,000**
*b*_3_	RB	LMS	−0.003	0.003	0.001	0.001	−0.044	0.008	0.002	0.001
		LMS_BOOT	−0.003	0.003	0.001	0.001	−0.044	0.008	0.002	0.001
		LMS_BAYES	−0.006	0.003	0.000	0.001	−0.071	0.002	−0.001	0.002
		PA_BOOT	−0.003	0.003	0.001	0.001	−0.005	0.003	0.000	0.000
		PA_BAYES	−0.002	0.003	0.001	0.001	−0.005	0.003	0.000	0.000
		CPI_BOOT	−0.002	0.003	0.001	0.001	−0.056	0.008	0.005	0.004
		UPI_BOOT	−0.005	0.003	0.001	0.001	−0.056	0.013	0.001	0.006
	SE/SD	LMS	0.928	0.989	0.973	1.007	**0.296**	1.076	1.0100	1.002
		LMS_BOOT	1.074	1.057	0.998	1.011	**0.486**	**1.325**	1.071	1.026
		LMS_BAYES	1.030	1.046	0.990	1.032	1.040	**1.644**	1.023	1.036
		PA_BOOT	1.000	1.039	0.982	1.008	0.992	1.069	0.992	0.996
		PA_BAYES	0.998	1.052	0.992	1.022	0.988	1.075	1.000	1.011
		CPI_BOOT	**1.167**	1.091	1.004	1.016	**1.832**	**3.188**	**1.317**	1.075
		UPI_BOOT	**1.822**	**1.226**	1.026	1.023	**2.390**	**2.841**	**2.088**	**2.162**
	Cov	LMS	0.930	0.944	0.947	0.953	0.961	0.969	0.957	0.954
		LMS_BOOT	0.958	0.960	0.945	0.95	0.995	0.989	0.959	0.957
		LMS_BAYES	0.960	0.957	0.949	0.965	0.962	0.961	0.950	0.964
		PA_BOOT	0.950	0.953	0.942	0.945	0.935	0.957	0.945	0.937
		PA_BAYES	0.958	0.962	0.954	0.959	0.952	0.965	0.952	0.951
		CPI_BOOT	0.979	0.954	0.950	0.951	0.988	0.999	0.991	0.971
		UPI_BOOT	0.993	0.977	0.960	0.955	0.997	1.000	1.000	0.999
	Type I	LMS	**0.070**	0.056	0.053	0.047	0.039	**0.031**	0.043	0.046
		LMS_BOOT	0.042	0.040	0.055	0.050	**0.005**	**0.011**	0.041	0.043
		LMS_BAYES	0.040	0.043	0.051	**0.035**	0.038	0.039	0.050	**0.036**
		PA_BOOT	0.050	0.047	0.058	0.055	**0.065**	0.043	0.055	0.063
		PA_BAYES	0.042	0.038	0.046	0.041	0.048	**0.035**	0.048	0.049
		CPI_BOOT	**0.021**	0.046	0.050	0.049	**0.011**	**0.001**	**0.009**	**0.029**
		UPI_BOOT	**0.007**	**0.023**	0.040	0.045	**0.003**	**0.000**	**0.000**	**0.001**
ind	RB	LMS	0.029	−0.002	0.000	0.001	**0.196**	−0.04	0.026	0.004
		LMS_BOOT	0.029	−0.002	0.000	0.001	**0.196**	−0.04	0.026	0.004
		LMS_BAYES	0.037	0.002	0.004	0.000	**0.123**	**0.153**	0.067	0.004
		PA_BOOT	**−0.263**	**−0.272**	**−0.270**	**−0.269**	**−0.641**	**−0.646**	**−0.646**	**−0.645**
		PA_BAYES	**−0.266**	**−0.273**	**−0.270**	**−0.268**	**−0.649**	**−0.649**	**−0.646**	**−0.645**
		CPI_BOOT	0.019	−0.004	−0.001	0.000	0.036	0.014	0.035	0.005
		UPI_BOOT	0.030	0.000	0.001	0.001	0.033	0.039	0.041	0.008
	SE/SD	LMS	0.921	1.017	0.996	0.996	**0.428**	**0.518**	0.941	1.031
		LMS_BOOT	**43.688**	**3.032**	1.020	1.001	**3,844.443**	**450.192**	**273.606**	**17.844**
		LMS_BAYES	**1.212**	**1.118**	1.020	1.003	**8.445**	**97.091**	**2.178**	**1.116**
		PA_BOOT	0.960	1.024	0.992	0.981	0.974	1.062	0.988	0.989
		PA_BAYES	0.990	1.043	1.000	0.995	1.013	1.086	1.000	1.007
		CPI_BOOT	**1.253**	1.092	1.020	1.004	**1.248**	**1.395**	**1.779**	**1.248**
		UPI_BOOT	**1.464**	**1.127**	1.025	1.005	**1.636**	**1.506**	**2.036**	**1.635**
	Cov	LMS	0.931	0.949	0.944	0.942	0.947	0.961	0.955	0.954
		LMS_BOOT	0.959	0.960	0.950	0.944	0.992	0.987	0.979	0.968
		LMS_BAYES	0.948	0.959	0.951	0.943	0.982	0.976	0.953	0.951
		PA_BOOT	**0.694**	**0.483**	**0.158**	**0.018**	**0.059**	**0.001**	**0.000**	**0.000**
		PA_BAYES	**0.754**	**0.540**	**0.185**	**0.022**	**0.084**	**0.001**	**0.000**	**0.000**
		CPI_BOOT	0.961	0.958	0.952	0.943	0.974	0.988	0.981	0.971
		UPI_BOOT	0.975	0.963	0.956	0.945	0.981	0.985	0.989	0.982
	Power	LMS	0.826	0.979	1.000	1.000	**0.085**	**0.334**	**0.790**	0.975
		LMS_BOOT	**0.661**	0.968	1.000	1.000	**0.013**	**0.059**	**0.488**	0.925
		LMS_BAYES	**0.741**	0.969	1.000	1.000	**0.071**	**0.235**	**0.708**	0.945
		PA_BOOT	0.961	1.000	1.000	1.000	**0.700**	0.961	1.000	1.000
		PA_BAYES	0.964	1.000	1.000	1.000	**0.769**	0.972	1.000	1.000
		CPI_BOOT	**0.632**	0.964	1.000	1.000	**0.027**	**0.037**	**0.419**	0.911
		UPI_BOOT	**0.560**	0.950	1.000	1.000	**0.020**	**0.033**	**0.284**	**0.758**
index	RB	LMS	−0.002	0.003	0.001	0.001	−0.011	0.006	0.001	0.001
		LMS_BOOT	−0.002	0.003	0.001	0.001	−0.011	0.006	0.001	0.001
		LMS_BAYES	−0.004	0.002	0.000	0.001	−0.019	0.001	−0.001	0.001
		PA_BOOT	−0.002	0.002	0.000	0.000	−0.002	0.001	0.000	0.000
		PA_BAYES	−0.001	0.002	0.001	0.000	−0.002	0.001	0.000	0.000
		CPI_BOOT	−0.001	0.002	0.001	0.001	−0.031	0.005	0.004	0.003
		UPI_BOOT	−0.003	0.002	0.001	0.001	−0.035	0.010	0.000	0.005
	SE/SD	LMS	0.925	0.985	0.974	1.010	0.999	1.073	1.005	1.003
		LMS_BOOT	1.064	1.050	0.997	1.014	**1,051.325**	**46.105**	1.069	1.030
		LMS_BAYES	1.045	1.051	0.994	1.033	**2.262**	**1.609**	1.036	1.040
		PA_BOOT	0.998	1.037	0.979	1.006	1.008	1.084	0.987	0.991
		PA_BAYES	1.022	1.062	0.991	1.031	1.062	**1.117**	1.007	1.009
		CPI_BOOT	**1.156**	1.081	1.003	1.018	**1.943**	**3.154**	**1.330**	1.080
		UPI_BOOT	**1.812**	**1.219**	1.023	1.026	**2.415**	**3.008**	**2.200**	**2.148**
	Cov	LMS	0.940	0.947	0.949	0.953	0.988	0.978	0.955	0.958
		LMS_BOOT	0.958	0.959	0.948	0.949	0.999	0.992	0.962	0.960
		LMS_BAYES	0.960	0.957	0.949	0.965	0.962	0.961	0.950	0.964
		PA_BOOT	0.953	0.958	0.947	0.945	0.957	0.967	0.952	0.938
		PA_BAYES	0.958	0.962	0.954	0.959	0.954	0.965	0.952	0.951
		CPI_BOOT	0.983	0.958	0.950	0.950	0.990	1.000	0.993	0.967
		UPI_BOOT	0.994	0.980	0.959	0.956	0.997	0.999	1.000	0.999
	Type I	LMS	0.060	0.053	0.051	0.047	**0.012**	**0.022**	0.045	0.042
		LMS_BOOT	0.042	0.041	0.052	0.051	**0.001**	**0.008**	0.038	0.040
		LMS_BAYES	0.040	0.043	0.051	**0.035**	0.038	0.039	0.050	**0.036**
		PA_BOOT	0.047	0.042	0.053	0.055	0.043	**0.033**	0.048	0.062
		PA_BAYES	0.042	0.038	0.046	0.041	0.046	**0.035**	0.048	0.049
		CPI_BOOT	**0.017**	0.042	0.050	0.050	**0.008**	**0.000**	**0.007**	**0.033**
		UPI_BOOT	**0.006**	**0.020**	0.041	0.044	**0.003**	**0.001**	**0.000**	**0.001**

b_3_, the effect of XZ on Y; ind, the average estimates of the indirect effects at different values of the moderator (a * (b_1_ + b_3_ * Z), whenZ = 0, 1SD, − 1SD); index, the moderated mediation index (a * b_3_); N, sample size.

*Estimator: LMS, Latent moderated structural equation model with robust standard error generated by maximum likelihood estimator; LMS_BOOT, Latent moderated structural equation model with bootstrapped standard error; LMS_BAYES, Latent moderated structural equation model with Bayesian estimation; PA_BOOT, Path analysis with bootstrapping method; PA_BAYES, Path analysis with Bayesian estimation; CPI_BOOT, Constrained product indicator model with bootstrapping method; UPI_BOOT, Unconstrained product indicator model with bootstrapping method; RB, parameter estimates (b_3_ = 0)/relative bias of the parameter estimates (b_3_ ≠ 0); Cov, 95% coverage; Type I, Type I error rate. Boldfaced values represent the ones that are out of the acceptable range of the evaluation criteria*.

**Table 2:** Results of Simulation Study 1 when *b*_3_ = 0.2.

***N***		**Estimator**	**High reliability**				**Low reliability**			
			**100**	**200**	**500**	**1,000**	**100**	**200**	**500**	**L,000**
*b*_3_	RB	LMS	−0.018	0.013	0.001	0.000	−0.047	0.042	−0.002	−0.002
		LMS_BOOT	−0.018	0.013	0.001	0.000	−0.047	0.042	−0.002	−0.002
		LMS_BAYES	0.006	0.029	0.005	0.006	0.034	**0.140**	0.029	0.022
		PA_BOOT	**−0.103**	−0.069	−0.083	−0.082	**−0.593**	**−0.548**	**−0.565**	**−0.564**
		PA_BAYES	−0.100	−0.067	−0.081	−0.081	**−0.590**	**−0.547**	**−0.563**	**−0.563**
		CPI_BOOT	0.009	0.024	0.006	0.005	**0.136**	**0.203**	0.057	0.032
		UPI_BOOT	0.013	0.023	0.008	0.006	**−0.110**	**0.423**	**0.309**	**0.119**
	SE/SD	LMS	0.937	0.992	0.963	1.003	**1.129**	1.046	0.994	1.006
		LMS_BOOT	1.076	1.056	0.984	1.007	**1.553**	**1.221**	1.049	1.028
		LMS_BAYES	1.037	1.052	0.979	1.034	**2.781**	**1.681**	1.005	1.050
		PA_BOOT	1.003	1.031	0.978	1.015	0.992	1.055	0.995	1.004
		PA_BAYES	0.993	1.034	0.975	1.015	0.978	1.050	0.990	1.004
		CPI_BOOT	**1.164**	1.077	0.992	1.016	**1.884**	**2.878**	**1.274**	1.079
		UPI_BOOT	**1.680**	**1.229**	1.016	1.038	**2.378**	**2.604**	**1.962**	**2.129**
	Cov	LMS	0.929	0.951	0.940	0.950	0.952	0.957	0.940	0.955
		LMS_BOOT	0.957	0.962	0.938	0.948	0.982	0.974	0.950	0.952
		LMS_BAYES	0.957	0.960	0.942	0.956	0.970	0.958	0.948	0.967
		PA_BOOT	0.940	0.950	0.917	0.912	**0.735**	**0.570**	**0.177**	**0.018**
		PA_BAYES	0.945	0.950	0.917	0.913	**0.727**	**0.568**	**0.169**	**0.018**
		CPI_BOOT	0.976	0.961	0.944	0.945	0.993	0.999	0.987	0.968
		UPI_BOOT	0.990	0.965	0.953	0.955	0.998	0.992	0.991	0.979
	Power	LMS	**0.545**	0.871	0.996	1.000	**0.132**	**0.352**	**0.745**	0.974
		LMS_BOOT	**0.431**	0.833	0.996	1.000	**0.030**	**0.227**	**0.704**	0.965
		LMS_BAYES	**0.498**	0.853	0.996	1.000	**0.158**	**0.382**	**0.767**	0.976
		PA_BOOT	**0.488**	0.837	0.992	1.000	**0.167**	**0.310**	**0.615**	0.885
		PA_BAYES	**0.486**	0.828	0.993	1.000	**0.159**	**0.289**	**0.609**	0.883
		CPI_BOOT	**0.309**	**0.740**	0.987	1.000	**0.008**	**0.004**	**0.195**	**0.626**
		UPI_BOOT	**0.176**	**0.604**	0.979	1.000	**0.002**	**0.004**	**0.015**	**0.117**
ind	RB	LMS	0.034	−0.002	0.000	0.001	0.027	−0.028	0.024	0.003
		LMS_BOOT	0.034	−0.002	0.000	0.001	0.027	−0.028	0.024	0.003
		LMS_BAYES	0.041	−0.001	0.005	0.000	**0.719**	**0.156**	0.077	0.004
		PA_BOOT	**−0.256**	**−0.269**	**−0.266**	**−0.264**	**−0.630**	**−0.638**	**−0.636**	**−0.636**
		PA_BAYES	**−0.260**	**−0.270**	**−0.266**	**−0.264**	**−0.639**	**−0.641**	**−0.637**	**−0.636**
		CPI_BOOT	0.023	−0.005	−0.002	−0.001	0.093	−0.017	0.031	−0.001
		UPI_BOOT	0.033	−0.001	−0.001	0.000	0.091	0.000	−0.002	−0.008
	SE/SD	LMS	0.925	1.014	0.991	0.994	1.099	**1.333**	0.947	1.019
		LMS_BOOT	**23.474**	1.093	1.014	0.999	**2,028.156**	**466.526**	**237.405**	**11.981**
		LMS_BAYES	**1.212**	**1.109**	1.016	1.000	**1.674**	**78.763**	**2.435**	**1.103**
		PA_BOOT	0.957	1.008	0.982	0.978	0.973	1.057	0.979	0.991
		PA_BAYES	0.985	1.022	0.985	0.986	1.009	1.079	0.986	1.002
		CPI_BOOT	**1.257**	1.085	1.007	1.001	**1.206**	**1.737**	**1.770**	**1.242**
		UPI_BOOT	**1.428**	**1.127**	1.013	1.005	**1.345**	**1.667**	**2.016**	**1.679**
	Cov	LMS	0.940	0.95	0.948	0.947	0.945	0.962	0.957	0.954
		LMS_BOOT	0.969	0.961	0.949	0.945	0.994	0.988	0.978	0.964
		LMS_BAYES	0.954	0.959	0.951	0.946	0.982	0.976	0.952	0.944
		PA_BOOT	**0.683**	**0.508**	**0.233**	**0.100**	**0.127**	**0.023**	**0.000**	**0.000**
		PA_BAYES	**0.747**	**0.559**	**0.253**	**0.112**	**0.155**	**0.034**	**0.000**	**0.000**
		CPI_BOOT	0.966	0.959	0.952	0.947	0.973	0.990	0.980	0.972
		UPI_BOOT	0.975	0.962	0.951	0.948	0.978	0.985	0.989	0.983
	Power	LMS	**0.748**	0.898	0.994	1.000	**0.100**	**0.356**	**0.714**	0.912
		LMS_BOOT	**0.611**	0.874	0.994	1.000	**0.010**	**0.076**	**0.468**	0.853
		LMS_BAYES	**0.680**	0.881	0.994	1.000	**0.075**	**0.231**	**0.661**	0.878
		PA_BOOT	0.893	0.977	1.000	1.000	**0.673**	0.910	0.997	1.000
		PA_BAYES	0.898	0.978	1.000	1.000	**0.734**	0.917	0.998	1.000
		CPI_BOOT	**0.594**	0.867	0.994	1.000	**0.024**	**0.035**	**0.402**	0.811
		UPI_BOOT	**0.524**	0.852	0.987	1.000	**0.021**	**0.041**	**0.283**	**0.679**
index	RB	LMS	−0.013	0.017	0.004	0.003	−0.026	0.045	0.009	0.006
		LMS_BOOT	−0.013	0.017	0.004	0.003	−0.026	0.045	0.009	0.006
		LMS_BAYES	0.001	0.030	0.003	0.009	0.071	**0.179**	0.032	0.037
		PA_BOOT	**−0.287**	**−0.257**	**−0.269**	**−0.268**	**−0.783**	**−0.761**	**−0.769**	**−0.770**
		PA_BAYES	**−0.292**	**−0.259**	**−0.267**	**−0.267**	**−0.789**	**−0.765**	**−0.770**	**−0.769**
		CPI_BOOT	0.007	0.026	0.007	0.006	**0.177**	**0.185**	0.068	0.038
		UPI_BOOT	0.021	0.029	0.011	0.009	−0.010	**0.424**	**0.321**	**0.127**
	SE/SD	LMS	0.931	0.992	0.955	1.004	**1.103**	1.046	0.995	0.991
		LMS_BOOT	1.061	1.053	0.976	1.009	**1,284.607**	**1.229**	1.052	1.014
		LMS_BAYES	1.042	1.050	0.976	1.027	**5.436**	**1.665**	1.026	1.018
		PA_BOOT	1.005	1.021	0.976	1.018	1.003	1.066	0.994	1.009
		PA_BAYES	1.016	1.034	0.976	1.024	1.032	1.080	0.994	1.009
		CPI_BOOT	**1.153**	1.078	0.989	1.025	**1.851**	**3.022**	**1.292**	1.079
		UPI_BOOT	**1.686**	**1.217**	1.010	1.043	**2.292**	**2.712**	**2.080**	**2.107**
	Cov	LMS	0.934	0.946	0.939	0.949	0.934	0.956	0.944	0.951
		LMS_BOOT	0.957	0.956	0.938	0.949	0.978	0.975	0.952	0.952
		LMS_BAYES	0.962	0.951	0.946	0.956	0.958	0.959	0.950	0.957
		PA_BOOT	**0.864**	**0.817**	**0.621**	**0.338**	**0.166**	**0.019**	**0.000**	**0.000**
		PA_BAYES	**0.876**	**0.830**	**0.625**	**0.346**	**0.197**	**0.021**	**0.000**	**0.000**
		CPI_BOOT	0.975	0.956	0.943	0.947	0.995	0.999	0.991	0.963
		UPI_BOOT	0.987	0.967	0.953	0.953	0.998	0.997	0.991	0.986
	Power	LMS	**0.520**	0.867	0.997	1.000	**0.067**	**0.303**	**0.731**	0.970
		LMS_BOOT	**0.410**	0.828	0.995	1.000	**0.013**	**0.181**	**0.686**	0.964
		LMS_BAYES	**0.498**	0.853	0.996	1.000	**0.157**	**0.382**	**0.767**	0.976
		PA_BOOT	**0.469**	0.830	0.992	1.000	**0.121**	**0.280**	**0.595**	0.877
		PA_BAYES	**0.486**	0.828	0.993	1.000	**0.156**	**0.289**	**0.609**	0.883
		CPI_BOOT	**0.302**	**0.740**	0.987	1.000	**0.007**	**0.007**	**0.172**	**0.621**
		UPI_BOOT	**0.148**	**0.589**	0.978	1.000	**0.002**	**0.004**	**0.012**	**0.107**

b_3_, the effect of XZ on Y; ind, the average estimates of the indirect effects at different values of the moderator (a * (b_1_ + b_3_ * Z), whenZ = 0, 1SD, − 1SD); index, the moderated mediation index (a * b_3_); N, sample size;.

*Estimator: LMS, Latent moderated structural equation model with robust standard error generated by maximum likelihood estimator; LMS_BOOT, Latent moderated structural equation model with bootstrapped standard error; LMS_BAYES, Latent moderated structural equation model with Bayesian estimation; PA_BOOT, Path analysis with bootstrapping method; PA_BAYES, Path analysis with Bayesian estimation; CPI_BOOT, Constrained product indicator model with bootstrapping method; UPI_BOOT, Unconstrained product indicator model with bootstrapping method*.

*RB, parameter estimates (b_3_ = 0)/relative bias of the parameter estimates (b_3_ ≠ 0); Cov, 95% coverage. Boldfaced values represent the ones that are out of the acceptable range of the evaluation criteria*.

**Table 3:** Results of Simulation Study 1 when *b*_3_ = 0.4.

***N***		**Estimator**	**High reliability**				**Low reliability**			
			**100**	**200**	**500**	**1,000**	**100**	**200**	**500**	**L,000**
*b*_3_	RB	LMS	−0.011	0.004	−0.002	−0.001	−0.017	0.016	−0.007	−0.003
		LMS_BOOT	−0.011	0.004	−0.002	−0.001	−0.017	0.016	−0.007	−0.003
		LMS_BAYES	0.021	0.022	0.005	0.004	**0.122**	**0.117**	0.030	0.020
		PA_BOOT	−0.097	−0.076	−0.084	−0.083	**−0.580**	**−0.555**	**−0.565**	**−0.564**
		PA_BAYES	−0.095	−0.076	−0.084	−0.083	**−0.578**	**−0.555**	**−0.564**	**−0.564**
		CPI_BOOT	0.019	0.018	0.004	0.003	**0.258**	**0.192**	0.047	0.023
		UPI_BOOT	0.030	0.021	0.007	0.005	**0.209**	**0.536**	**0.180**	0.079
	SE/SD	LMS	0.950	0.997	0.962	1.006	1.071	1.009	0.986	1.006
		LMS_BOOT	1.075	1.050	0.975	1.009	**1.938**	**1.210**	1.040	1.015
		LMS_BAYES	1.046	1.055	0.975	1.034	**2.996**	**1.359**	1.000	1.057
		PA_BOOT	1.000	1.015	0.974	1.014	0.986	1.036	0.995	1.007
		PA_BAYES	0.965	0.989	0.937	0.979	0.949	0.998	0.959	0.972
		CPI_BOOT	**1.146**	1.058	0.987	1.014	**1.736**	**2.360**	**1.264**	1.076
		UPI_BOOT	**1.675**	**1.203**	1.011	1.034	**1.637**	**1.113**	**2.562**	**1.805**
	Cov	LMS	0.928	0.949	0.930	0.949	0.936	0.941	0.938	0.956
		LMS_BOOT	0.955	0.956	0.933	0.947	0.976	0.966	0.949	0.946
		LMS_BAYES	0.947	0.959	0.928	0.959	0.966	0.954	0.948	0.961
		PA_BOOT	0.924	0.921	**0.860**	**0.784**	**0.333**	**0.094**	**0.000**	**0.000**
		PA_BAYES	0.923	0.915	**0.842**	**0.759**	**0.306**	**0.068**	**0.000**	**0.000**
		CPI_BOOT	0.966	0.958	0.938	0.941	0.991	0.994	0.977	0.963
		UPI_BOOT	0.986	0.964	0.951	0.948	0.987	0.984	0.976	0.971
	Power	LMS	0.953	1.000	1.000	1.000	**0.372**	0.829	0.997	1.000
		LMS_BOOT	0.923	1.000	1.000	1.000	**0.153**	**0.719**	0.995	1.000
		LMS_BAYES	0.953	1.000	1.000	1.000	**0.524**	0.871	0.999	1.000
		PA_BOOT	0.932	1.000	1.000	1.000	**0.426**	**0.759**	0.984	1.000
		PA_BAYES	0.939	1.000	1.000	1.000	**0.434**	**0.777**	0.984	1.000
		CPI_BOOT	0.815	0.996	1.000	1.000	**0.011**	**0.031**	**0.715**	0.997
		UPI_BOOT	**0.500**	0.959	1.000	1.000	**0.012**	**0.010**	**0.072**	**0.486**
ind	RB	LMS	0.060	−0.003	0.000	0.001	**0.139**	0.079	0.020	0.001
		LMS_BOOT	0.060	−0.003	0.000	0.001	**0.139**	0.079	0.020	0.001
		LMS_BAYES	0.054	−0.015	0.006	−0.004	**0.308**	**0.220**	**0.109**	−0.003
		PA_BOOT	**−0.216**	**−0.246**	**−0.239**	**−0.237**	**−0.567**	**−0.583**	**−0.579**	**−0.579**
		PA_BAYES	**−0.228**	**−0.252**	**−0.241**	**−0.238**	**−0.580**	**−0.589**	**−0.581**	**−0.579**
		CPI_BOOT	0.043	−0.013	−0.004	−0.003	−0.084	**−0.114**	0.017	−0.019
		UPI_BOOT	0.040	−0.013	−0.006	−0.005	**−0.168**	**−0.295**	−0.084	−0.060
	SE/SD	LMS	0.932	1.010	0.987	0.991	**1.740**	**1.193**	0.937	1.011
		LMS_BOOT	**29.612**	1.084	1.008	0.996	**1,268**	**435.094**	**240.396**	**13.104**
		LMS_BAYES	**1.203**	1.100	1.011	0.996	**5.692**	**90.189**	**2.469**	1.088
		PA_BOOT	0.946	0.984	0.961	0.965	0.969	1.046	0.969	0.989
		PA_BAYES	0.967	0.988	0.955	0.961	1.001	1.058	0.967	0.990
		CPI_BOOT	**1.257**	1.069	0.989	0.990	**1.290**	**1.804**	**1.802**	**1.230**
		UPI_BOOT	**1.416**	**1.116**	0.994	0.995	**1.219**	**1.407**	**2.233**	**1.551**
	Cov	LMS	0.941	0.947	0.946	0.943	0.949	0.965	0.959	0.956
		LMS_BOOT	0.972	0.959	0.949	0.946	0.992	0.988	0.975	0.968
		LMS_BAYES	0.957	0.958	0.950	0.943	0.984	0.981	0.955	0.946
		PA_BOOT	**0.689**	**0.528**	**0.347**	**0.292**	**0.293**	**0.250**	**0.174**	**0.09**
		PA_BAYES	**0.734**	**0.561**	**0.356**	**0.297**	**0.306**	**0.266**	**0.199**	**0.102**
		CPI_BOOT	0.967	0.955	0.948	0.942	0.973	0.990	0.979	0.974
		UPI_BOOT	0.974	0.960	0.949	0.946	0.973	0.986	0.989	0.980
	Power	LMS	**0.658**	**0.722**	0.812	0.905	**0.132**	**0.385**	**0.624**	**0.723**
		LMS_BOOT	**0.562**	**0.710**	0.806	0.901	**0.013**	**0.086**	**0.445**	**0.692**
		LMS_BAYES	**0.611**	**0.710**	0.807	0.897	**0.088**	**0.222**	**0.584**	**0.715**
		PA_BOOT	**0.742**	**0.791**	0.911	0.985	**0.620**	**0.790**	0.923	0.990
		PA_BAYES	**0.736**	**0.786**	0.911	0.988	**0.674**	**0.790**	0.924	0.989
		CPI_BOOT	**0.554**	**0.707**	0.800	0.891	**0.027**	**0.042**	**0.404**	**0.673**
		UPI_BOOT	**0.506**	**0.696**	**0.786**	0.871	**0.028**	**0.030**	**0.255**	**0.61**
index	RB	LMS	−0.006	0.008	0.000	0.002	−0.002	0.020	0.004	0.004
		LMS_BOOT	−0.006	0.008	0.000	0.002	−0.002	0.020	0.004	0.004
		LMS_BAYES	0.017	0.024	0.002	0.007	**0.266**	**0.176**	0.037	0.035
		PA_BOOT	**−0.281**	**−0.264**	**−0.270**	**−0.269**	**−0.777**	**−0.765**	**−0.770**	**−0.770**
		PA_BAYES	**−0.286**	**−0.266**	**−0.270**	**−0.268**	**−0.783**	**−0.768**	**−0.770**	**−0.770**
		CPI_BOOT	0.015	0.020	0.005	0.004	**0.296**	**0.179**	0.058	0.029
		UPI_BOOT	0.035	0.025	0.009	0.008	**0.332**	**0.535**	**0.195**	0.087
	SE/SD	LMS	0.948	0.998	0.954	0.996	**1.162**	1.034	0.991	0.983
		LMS_BOOT	1.062	1.049	0.967	0.996	**381.764**	**2.040**	1.048	1.001
		LMS_BAYES	1.044	1.046	0.972	1.011	**1.516**	**1.332**	1.024	0.989
		PA_BOOT	1.006	1.002	0.975	1.021	0.995	1.040	1.006	1.008
		PA_BAYES	0.989	0.984	0.940	0.989	0.986	1.011	0.966	0.976
		CPI_BOOT	**1.145**	1.067	0.981	1.018	**1.634**	**2.666**	**1.278**	1.070
		UPI_BOOT	**1.657**	**1.188**	1.002	1.034	**1.483**	**1.218**	**2.541**	**1.763**
	Cov	LMS	0.923	0.940	0.927	0.949	0.911	0.937	0.947	0.941
		LMS_BOOT	0.950	0.943	0.934	0.944	0.966	0.950	0.950	0.943
		LMS_BAYES	0.958	0.947	0.945	0.952	0.964	0.949	0.947	0.942
		PA_BOOT	**0.690**	**0.545**	**0.181**	**0.020**	**0.013**	**0.000**	**0.000**	**0.000**
		PA_BAYES	**0.718**	**0.569**	**0.178**	**0.016**	**0.016**	**0.000**	**0.000**	**0.000**
		CPI_BOOT	0.967	0.957	0.940	0.945	0.993	0.996	0.98	0.965
		UPI_BOOT	0.980	0.966	0.952	0.954	0.987	0.987	0.977	0.973
	Power	LMS	0.950	1.000	1.000	1.000	**0.243**	**0.787**	0.996	1.000
		LMS_BOOT	0.913	1.000	1.000	1.000	**0.080**	**0.632**	0.995	1.000
		LMS_BAYES	0.953	1.000	1.000	1.000	**0.524**	0.871	0.999	1.000
		PA_BOOT	0.930	0.999	1.000	1.000	**0.310**	**0.722**	0.982	1.000
		PA_BAYES	0.939	1.000	1.000	1.000	**0.430**	**0.777**	0.984	1.000
		CPI_BOOT	0.800	0.996	1.000	1.000	**0.009**	**0.023**	**0.696**	0.997
		UPI_BOOT	**0.467**	0.959	1.000	1.000	**0.012**	**0.005**	**0.056**	**0.463**

b_3_, the effect of XZ on Y; ind, the average estimates of the indirect effects at different values of the moderator (a * (b_1_ + b_3_ * Z), whenZ = 0, 1SD, − 1SD); index, the moderated mediation index (a * b_3_); N, sample size.

*Estimator: LMS, Latent moderated structural equation model with robust standard error generated by maximum likelihood estimator; LMS_BOOT, Latent moderated structural equation model with bootstrapped standard error; LMS_BAYES, Latent moderated structural equation model with Bayesian estimation; PA_BOOT, Path analysis with bootstrapping method; PA_BAYES, Path analysis with Bayesian estimation; CPI_BOOT, Constrained product indicator model with bootstrapping method; UPI_BOOT, Unconstrained product indicator model with bootstrapping method; RB, parameter estimates (b_3_ = 0)/relative bias of the parameter estimates (b_3_ ≠ 0); Cov, 95% coverage. Boldfaced values represent the ones that are out of the acceptable range of the evaluation criteria*.

Moreover, when reliability is high, LMS, CPI, and UPI provide smaller estimate biases than PA, and the estimated values using these three approaches are expected to become more precise as the sample size increases. Under the condition of low reliability, although CPI and UPI presented biased estimates in many situations, CPI was more suitable than UPI overall. However, as demonstrated by previous research (Marsh et al., 2004), when sample size was small (*N* = 100), UPI was less biased than CPI when reliability was low.

Compared with the other three approaches, LMS always resulted in a lower relative bias of the estimates, especially with low reliability. LMS with ML estimation provided more accurate estimates than the Bayesian estimation under most conditions. However, LMS with Bayesian estimation performed best among all the approaches in many conditions when the sample size was 100 and *b*_3_ ≠ 0.

#### SE Ratio

The SE ratios of LMS with robust standard errors and PA were all within the acceptable interval of [0.9, 1.1] when the reliability is high, which demonstrates a relatively accurate estimate. This result is superior to the results for CPI and UPI, which both could not provide a relatively precise estimate of the SE. With low reliability, these two approaches can also provide an acceptable SE ratio under most conditions. Both CPI and UPI had SE ratios that fell outside of the standard interval of [0.9, 1.1].

Moreover, LMS with the bootstrap method seriously overestimated the standard errors of indirect effects when the sample size was 100 or reliability was low, which can be circumvented by the robust standard error. LMS with Bayesian estimation performed better than the bootstrap method in these conditions; however, it still overestimated standard errors.

#### Coverage Rate

The coverage rates of the PA decreased as the sample size grew. When the sample size is no less than 500 or the parameters of interest are the moderated mediation index and the indirect effects, PA cannot achieve acceptable coverage rates in most conditions. LMS, CPI and UPI could fit the 90% coverage rate when reliability is high. Under low reliability conditions, PI and LMS had acceptable coverage rates in most conditions, but PI performed better than LMS because PI produced overestimates of SE.

#### Power

Results show that all four approaches would have a higher power with an increased sample size (*N*), path coefficient of interaction (*b*_3_), and reliability. The statistical power of LMS and PA was found to be higher than that of CPI, and the statistical power of CPI was higher than that of UPI across all conditions. When reliability is low, all the approaches would all be less capable of providing an acceptable power of *b*_3_ and moderation effect when the sample size is low (*N* = 100 and *N* = 200). Considering indirect effects, PA performed best and still provided acceptable power even with low reliability while other approaches failed.

#### Type I Error Rate

When *b*_3_ = 0, the four approaches can be compared with regards to the type I error. Table 1 shows that the type I error of the four approaches is between [0.006, 0.070] and [0, 0.063] when the reliability is high or low, respectively. Of the four approaches, PA and LMS had a relatively larger type I error, and UPI resulted in the lowest type I error. In three LMS modeling conditions, LMS with the bootstrap method performed best when the reliability was low since it often overestimated the standard error.

#### Completion Rate

Table 4 shows the completion rate of these approaches under different conditions. PA had a 100% completion rate across all conditions. Additionally, LMS and CPI had acceptable completion rates, whereas UPI had the lowest completion rate when the conditions involved low reliability and relatively small sample size (*N* = 100 and 200).

**Table 4:** Completion rate of the four approaches in study 1.

	**Estimator**	**High reliability**				**Low reliability**			
***b*_3_**	**N**	**100**	**200**	**500**	**1,000**	**100**	**200**	**500**	**1,000**
0	LMS	0.999	1	1	1	0.926	0.982	1	1
	LMS_BOOT	0.999	1	1	1	0.933	0.982	1	1
	LMS_BAYES	1	1	1	1	0.716	0.976	1	1
	PA_BOOT	1	1	1	1	1	1	1	1
	PA_BAYES	1	1	1	1	1	1	1	1
	CPI_BOOT	1	1	1	1	0.944	0.991	1	1
	UPI_BOOT	0.99	1	1	1	0.597	0.672	0.826	0.93
0.2	LMS	1	1	1	1	0.925	0.987	1	1
	LMS_BOOT	1	1	1	1	0.925	0.987	1	1
	LMS_BAYES	1	1	1	1	0.739	0.982	1	1
	PA_BOOT	1	1	1	1	1	1	1	1
	PA_BAYES	1	1	1	1	1	1	1	1
	CPI_BOOT	1	1	1	1	0.946	0.99	1	1
	UPI_BOOT	0.996	1	1	1	0.626	0.732	0.904	0.98
0.4	LMS	1	1	1	1	0.923	0.987	1	1
	LMS_BOOT	1	1	1	1	0.923	0.987	1	1
	LMS_BAYES	1	1	1	1	0.754	0.977	1	1
	PA_BOOT	1	1	1	1	1	1	1	1
	PA_BAYES	1	1	1	1	1	1	1	1
	CPI_BOOT	1	1	1	1	0.941	0.99	1	1
	UPI_BOOT	1	1	1	1	0.682	0.83	0.986	0.998

*b_3_, the effect of XZ on Y; N, sample size*.

*Estimator: LMS, Latent moderated structural equation model with robust standard error generated by maximum likelihood estimator; LMS_BOOT, Latent moderated structural equation model with bootstrapped standard error; LMS_BAYES, Latent moderated structural equation model with Bayesian estimation; PA_BOOT, Path analysis with bootstrapping method; PA_BAYES, Path analysis with Bayesian estimation; CPI_BOOT, Constrained product indicator model with bootstrapping method; UPI_BOOT, Unconstrained product indicator model with bootstrapping method*.

## Study 2

Previous research has taken the violation of the normality assumption, and the influence this has on the estimations of the path coefficient of the interaction effect, into account (e.g., Marsh et al., 2004). Although the indicators of M and Z are normally distributed, the product indicators of MZ and the latent interaction of MZ may not obey the assumption of multivariate normality (Klein and Moosbrugger, 2000). This condition would result in a biased estimation of the SE, significance testing, and confidence interval, which are all based on the multivariate normally distributed assumption. Study 1 and previous research (Marsh et al., 2004) have proven that PIs can provide unbiased estimates when using normally distributed data. However, if the indicators of M and Z are non-normally distributed, CPI is found to result in more biased estimates than UPI (Marsh et al., 2004). For indicators that are slightly non-normal (e.g., skewness = 0.5 and kurtosis = 1.1), LMS's estimates are still within the acceptable interval (Klein and Moosbrugger, 2000; Klein and Muthén, 2007), and have stronger testing power (Cham et al., 2012). Nevertheless, for the indicators that are strongly non-normal (e.g., skewness = 0.9 and kurtosis = 1.5), researchers have found that both LMS and CPI would be biased in estimating the path coefficient of the interaction effect (Coenders et al., 2008). In study 2, we explore the effects on the estimates of the four approaches with varying non-normally distributed data.

In study 1, we neglected the possibility that X and Z were correlated. However, this is likely in practical situations, particularly in the moderated mediation model. Therefore, in study 2, we also compared the estimates based on the four approaches when X and Z were related. In other words, the correlation of X and Z was set at 0 / 0.3.

### Population Model

The data settings in study 2 are similar to those in study 1 regarding the factor loadings, error variances of indicators, variances of latent variables, and path coefficients of *a, b*_1_, *b*_2_, and *c*. To simplify, only high reliability conditions will be considered in study 2. Moreover, we will only consider *b*_3_ = 0 and 0.2 for the path coefficient of the interaction effect with *N* = 200 (which represents a common, medium sample size in psychology research, Boomsma, 1982).

### Distribution of Indicators

This study controlled the distribution of errors (δ) for the indicators of M and Z to create non-normally distributed data. There were five types of distributions for the data:

Type 1: Normal distribution: δ_*m*_s, δ_*z*_s ~ *N*(0, 0.36).
Type 2: Uniform distribution: δ_*m*_s, δ_*z*_s ~ U[0, 1].
Type 3: Symmetrical moderated kurtosis distribution: δ_*m*_s, δ_*z*_s ~ $\frac{t_{5}}{\sqrt{\chi_{5}^{2}/3}}$ (Hu and Bentler, 1998).
Type 4: Symmetrical high kurtosis distribution: δ_*m*_s, δ_*z*_s ~ $\frac{K1}{\sqrt{\chi_{5}^{2}/3}}$ (Cham et al., 2012).
Type 5: Slightly skewed distribution: δ_*m*_s, δ_*z*_s ~ $\chi_{1}^{2}$.

The distribution of errors (δ) for the indicators of X and Y were still normally distributed as in study 1.

### Results

Based on the five distributions of data, the estimates of different effects are shown in Tables 5, 6.

**Table 5:** Results of Simulation Study 2 when *b*_3_ = 0.

		**Estimator**	**Cor** **=** **0**					**Cor** **=** **0.3**				
		**Type**	**1**	**2**	**3**	**4**	**5**	**1**	**2**	**3**	**4**	**5**
*b*_3_	RB	LMS	0.003	−0.001	−0.002	−0.001	0.032	0.000	−0.001	0.001	−0.002	0.017
		LMS_BOOT	0.003	−0.001	−0.002	−0.001	0.032	0.000	−0.001	0.001	−0.002	0.017
		LMS_BAYES	0.003	−0.001	−0.008	0.047	0.037	−0.001	−0.002	−0.002	0.023	0.017
		PA_BOOT	0.003	−0.001	0.000	−0.001	−0.001	0.001	−0.002	−0.001	−0.002	0.002
		PA_BAYES	0.003	−0.001	0.001	−0.001	0.000	0.001	−0.001	−0.001	−0.002	0.003
		CPI_BOOT	0.003	0.000	−0.001	−0.005	0.030	−0.001	−0.002	−0.004	0.018	0.034
		UPI_BOOT	0.003	0.000	−0.021	−0.080	0.032	−0.001	−0.002	−0.003	−0.007	0.038
	SE/SD	LMS	0.989	0.903	0.993	0.968	0.986	0.932	0.928	0.965	0.918	0.934
		LMS_BOOT	1.057	0.949	**1.196**	**1.372**	**1.260**	1.004	0.988	**1.147**	**1.274**	**1.196**
		LMS_BAYES	1.046	0.954	**3.537**	**7.028**	**6.891**	0.982	0.979	**1.742**	**10.659**	**1.849**
		PA_BOOT	1.039	0.937	1.015	0.922	0.979	0.994	0.976	0.978	0.910	0.971
		PA_BAYES	1.052	0.954	0.975	**0.737**	0.904	1.011	1.000	0.949	**0.738**	0.906
		CPI_BOOT	1.091	0.980	**2.052**	1.084	**1.523**	1.072	1.024	**1.908**	0.992	**1.593**
		UPI_BOOT	**1.226**	0.982	**2.716**	**1.589**	**1.484**	**1.162**	1.031	**2.607**	**1.938**	**2.074**
	Cov	LMS	0.944	0.918	0.953	0.927	0.917	0.926	0.924	0.951	0.937	0.931
		LMS_BOOT	0.960	0.926	0.982	0.980	0.972	0.946	0.941	0.974	0.983	0.967
		LMS_BAYES	0.957	0.941	0.947	0.928	0.911	0.947	0.944	0.948	0.925	0.918
		PA_BOOT	0.953	0.927	0.945	0.909	0.942	0.936	0.935	0.929	0.911	0.937
		PA_BAYES	0.962	0.940	0.946	**0.829**	0.933	0.957	0.948	0.925	**0.831**	0.931
		CPI_BOOT	0.954	0.939	0.999	**0.896**	0.983	0.962	0.950	0.997	0.928	0.989
		UPI_BOOT	0.977	0.945	0.998	0.993	0.995	0.976	0.951	1.000	0.998	0.998
	Type I	LMS	0.056	**0.082**	0.047	**0.073**	**0.083**	**0.074**	**0.076**	0.049	0.063	**0.069**
		LMS_BOOT	0.040	**0.074**	**0.018**	**0.020**	**0.028**	0.054	0.059	**0.026**	**0.017**	**0.033**
		LMS_BAYES	0.043	0.059	0.053	**0.072**	**0.089**	0.053	0.056	0.052	**0.075**	**0.082**
		PA_BOOT	0.047	**0.073**	0.055	**0.091**	0.058	**0.064**	**0.065**	**0.071**	**0.089**	0.063
		PA_BAYES	0.038	0.060	0.054	**0.171**	**0.067**	0.043	0.052	**0.075**	**0.169**	**0.069**
		CPI_BOOT	0.046	0.061	**0.001**	**0.080**	**0.013**	0.038	0.050	**0.002**	0.051	**0.010**
		UPI_BOOT	**0.023**	0.055	**0.002**	**0.007**	**0.005**	**0.024**	0.049	**0.000**	**0.002**	**0.002**
ind	RB	LMS	−0.002	−0.005	0.076	**0.116**	**0.131**	0.009	−0.008	0.065	**0.118**	**0.130**
		LMS_BOOT	−0.002	−0.005	0.076	**0.116**	**0.131**	0.009	−0.008	0.065	**0.118**	**0.129**
		LMS_BAYES	0.002	−0.005	**0.150**	**0.162**	**0.177**	0.012	−0.006	**0.125**	**0.126**	**0.185**
		PA_BOOT	**−0.272**	−0.085	**−0.621**	**−0.770**	**−0.667**	**−0.265**	−0.089	**−0.613**	**−0.764**	**−0.649**
		PA_BAYES	**−0.273**	−0.086	**−0.623**	**−0.772**	**−0.668**	**−0.269**	−0.093	**−0.617**	**−0.768**	**−0.654**
		CPI_BOOT	−0.004	−0.009	**0.102**	**0.296**	**0.161**	0.005	−0.011	0.078	**0.231**	**0.166**
		UPI_BOOT	0.000	−0.005	**0.123**	**0.403**	**0.177**	0.010	−0.008	**0.133**	**0.264**	**0.229**
	SE/SD	LMS	1.017	0.971	0.932	1.006	**0.751**	0.970	0.954	0.902	0.933	0.943
		LMS_BOOT	**3.032**	1.020	**85.708**	**140.314**	**133.429**	1.045	1.002	**109.736**	**134.64**	**140.4**
		LMS_BAYES	**1.118**	1.030	**10.499**	**58.322**	**19.048**	1.035	1.009	**13.459**	**30.292**	**19.215**
		PA_BOOT	1.024	0.987	0.992	0.913	0.947	1.003	0.975	0.953	**0.849**	0.995
		PA_BAYES	1.043	0.999	0.982	**0.782**	0.924	1.023	0.988	0.944	**0.724**	0.965
		CPI_BOOT	1.092	1.025	**1.699**	1.059	**1.537**	1.046	1.010	**1.611**	0.908	**1.248**
		UPI_BOOT	**1.127**	1.029	**1.966**	**1.143**	**1.299**	1.068	1.013	0.952	**1.106**	**1.453**
	Cov	LMS	0.949	0.938	0.944	0.908	0.924	0.945	0.938	0.938	0.912	0.944
		LMS_BOOT	0.960	0.951	0.972	0.965	0.963	0.957	0.946	0.964	0.965	0.972
		LMS_BAYES	0.959	0.953	0.969	0.983	0.959	0.957	0.948	0.969	0.980	0.972
		PA_BOOT	**0.483**	**0.890**	**0.006**	**0.006**	**0.003**	**0.505**	**0.877**	**0.010**	**0.007**	**0.005**
		PA_BAYES	**0.540**	0.907	**0.007**	**0.003**	**0.004**	**0.547**	**0.899**	**0.011**	**0.004**	**0.005**
		CPI_BOOT	0.958	0.949	0.982	0.900	0.969	0.957	0.948	0.976	0.903	0.977
		UPI_BOOT	0.963	0.950	0.988	0.967	0.978	0.964	0.947	0.981	0.975	0.989
	Power	LMS	0.979	0.994	**0.637**	**0.325**	**0.505**	0.978	0.990	**0.642**	**0.324**	**0.533**
		LMS_BOOT	0.968	0.991	**0.361**	**0.159**	**0.275**	0.967	0.986	**0.391**	**0.187**	**0.298**
		LMS_BAYES	0.969	0.991	**0.744**	**0.512**	**0.640**	0.969	0.984	**0.727**	**0.498**	**0.655**
		PA_BOOT	1.000	1.000	0.905	**0.546**	0.842	1.000	1.000	0.912	**0.560**	0.872
		PA_BAYES	1.000	1.000	0.926	**0.699**	0.881	0.999	1.000	0.933	**0.699**	0.900
		CPI_BOOT	0.964	0.991	**0.163**	**0.130**	**0.098**	0.969	0.987	**0.215**	**0.128**	**0.144**
		UPI_BOOT	0.950	0.991	**0.139**	**0.069**	**0.098**	0.961	0.986	**0.192**	**0.072**	**0.138**
index	RB	LMS	0.003	−0.001	−0.002	−0.001	0.023	0.000	−0.001	0.000	−0.001	0.012
		LMS_BOOT	0.003	−0.001	−0.002	−0.001	0.023	0.000	−0.001	0.000	−0.001	0.012
		LMS_BAYES	0.002	−0.001	−0.006	0.012	0.026	−0.001	−0.001	−0.002	0.020	0.012
		PA_BOOT	0.002	−0.001	0.000	−0.001	0.000	0.000	−0.001	−0.001	−0.001	0.001
		PA_BAYES	0.002	−0.001	0.000	−0.001	0.000	0.001	−0.001	0.000	−0.001	0.001
		CPI_BOOT	0.002	0.000	−0.002	−0.004	0.015	−0.001	−0.001	−0.003	0.011	0.022
		UPI_BOOT	0.002	0.000	−0.012	−0.058	0.016	−0.001	−0.001	−0.002	−0.010	0.029
	SE/SD	LMS	0.985	0.904	1.010	0.983	0.991	0.931	0.928	0.965	0.943	0.952
		LMS_BOOT	1.050	0.952	**1.171**	**1.186**	**1.172**	1.001	0.989	**1.111**	**1.156**	**1.140**
		LMS_BAYES	1.051	0.963	**3.596**	**6.427**	**6.729**	0.988	0.986	**1.653**	**5.396**	**1.700**
		PA_BOOT	1.037	0.934	1.014	0.920	0.972	0.992	0.972	0.980	0.920	0.976
		PA_BAYES	1.062	0.960	0.993	**0.765**	0.916	1.019	1.008	0.972	**0.779**	0.929
		CPI_BOOT	1.081	0.980	**2.138**	**1.224**	**1.499**	1.063	1.022	**1.849**	0.990	**1.611**
		UPI_BOOT	**1.219**	0.987	**2.783**	**1.512**	**1.417**	**1.157**	1.029	**2.624**	**1.789**	**2.141**
	Cov	LMS	0.947	0.921	0.949	0.906	0.921	0.929	0.929	0.944	0.925	0.928
		LMS_BOOT	0.959	0.932	0.976	0.971	0.968	0.945	0.944	0.969	0.975	0.958
		LMS_BAYES	0.957	0.941	0.947	0.928	0.911	0.947	0.944	0.948	0.925	0.918
		PA_BOOT	0.958	0.933	0.947	0.928	0.946	0.937	0.938	0.936	0.927	0.950
		PA_BAYES	0.962	0.940	0.946	**0.831**	0.933	0.957	0.948	0.925	**0.834**	0.931
		CPI_BOOT	0.958	0.943	0.999	0.904	0.981	0.962	0.950	0.996	0.933	0.987
		UPI_BOOT	0.980	0.948	0.998	0.991	0.993	0.979	0.952	1.000	0.998	0.998
	Type I	LMS	0.053	**0.079**	0.051	**0.094**	**0.079**	**0.071**	**0.071**	0.056	**0.075**	**0.072**
		LMS_BOOT	0.041	**0.068**	**0.024**	**0.029**	**0.032**	0.055	0.056	**0.031**	**0.025**	0.042
		LMS_BAYES	0.043	0.059	0.053	**0.072**	**0.089**	0.053	0.056	0.052	**0.075**	**0.082**
		PA_BOOT	0.042	**0.067**	0.053	**0.072**	0.054	0.063	0.062	**0.064**	**0.073**	0.050
		PA_BAYES	0.038	0.060	0.054	**0.169**	**0.067**	0.043	0.052	**0.075**	**0.166**	**0.069**
		CPI_BOOT	0.042	0.057	**0.001**	**0.071**	**0.014**	0.038	0.050	**0.003**	0.046	**0.012**
		UPI_BOOT	**0.020**	0.052	**0.002**	**0.009**	**0.007**	**0.021**	0.048	**0.000**	**0.002**	**0.002**

Cor, correlation between X and Z; b_3_, the effect of XZ on Y; ind, the average estimates of the indirect effects at different values of the moderator (a * (b_1_ + b_3_ * Z), whenZ = 0, 1SD, − 1SD); index, the moderated mediation index (a * b_3_).

*Estimator: LMS, Latent moderated structural equation model with robust standard error generated by maximum likelihood estimator; LMS_BOOT, Latent moderated structural equation model with bootstrapped standard error; LMS_BAYES, Latent moderated structural equation model with Bayesian estimation; PA_BOOT, Path analysis with bootstrapping method; PA_BAYES, Path analysis with Bayesian estimation; CPI_BOOT, Constrained product indicator model with bootstrapping method; UPI_BOOT, Unconstrained product indicator model with bootstrapping method*.

*Type: Type 1, Normal distribution; Type 2, Uniform distribution; Type 3, Symmetrical moderated kurtosis distribution; Type 4, Symmetrical high kurtosis distribution; Type 5, Slightly skewed distribution; RB, parameter estimates (b_3_ = 0)/relative bias of the parameter estimates (b_3_ ≠ 0); Cov, 95% coverage; Type I, Type I error rate. Boldfaced values represent the ones that are out of the acceptable range of the evaluation criteria*.

**Table 6:** Results of Simulation Study 2 when *b*_3_ = 0.2.

		**Estimator**	**Cor** **=** **0**					**Cor** **=** **0.3**				
		**Type**	**1**	**2**	**3**	**4**	**5**	**1**	**2**	**3**	**4**	**5**
*b*_3_	RB	LMS	0.013	−0.005	0.032	**0.131**	**0.174**	−0.003	−0.007	0.032	**0.101**	**0.110**
		LMS_BOOT	0.013	−0.005	0.032	**0.131**	**0.174**	−0.003	−0.007	0.032	**0.101**	**0.110**
		LMS_BAYES	0.029	−0.004	**0.143**	**4.782**	**0.377**	0.012	−0.008	**0.146**	**1.210**	**0.550**
		PA_BOOT	−0.069	**0.150**	**−0.586**	**−0.788**	**−0.657**	−0.070	**0.150**	**−0.585**	**−0.783**	**−0.628**
		PA_BAYES	−0.067	**0.151**	**−0.585**	**−0.788**	**−0.656**	−0.066	**0.154**	**−0.583**	**−0.781**	**−0.626**
		CPI_BOOT	0.024	−0.003	**0.329**	**0.756**	**0.742**	−0.049	−0.052	−0.049	0.034	**0.221**
		UPI_BOOT	0.023	−0.003	**0.437**	**−0.189**	**0.932**	−0.058	−0.052	**−0.194**	**0.101**	**0.104**
	SE/SD	LMS	0.992	0.902	0.988	**1.170**	0.983	0.939	0.928	0.999	1.017	0.977
		LMS_BOOT	1.056	0.950	**1.169**	**1.395**	**1.326**	1.006	0.978	**1.188**	**1.582**	**1.254**
		LMS_BAYES	1.052	0.952	**2.349**	**1.853**	**3.254**	0.994	0.978	**1.385**	**2.130**	0.918
		PA_BOOT	1.031	0.937	1.004	0.918	0.961	0.994	0.972	0.964	**0.897**	0.972
		PA_BAYES	1.034	0.951	0.937	**0.710**	**0.868**	0.998	0.993	0.909	**0.704**	**0.874**
		CPI_BOOT	1.077	0.973	**1.848**	1.095	**1.341**	1.064	1.005	**1.758**	0.997	**1.220**
		UPI_BOOT	**1.229**	0.973	**1.587**	**1.496**	**1.392**	**1.142**	1.007	**2.493**	**1.283**	**1.564**
	Cov	LMS	0.951	0.917	0.958	0.952	0.957	0.930	0.919	0.951	0.943	0.954
		LMS_BOOT	0.962	0.934	0.976	0.984	0.980	0.945	0.940	0.973	0.980	0.975
		LMS_BAYES	0.960	0.941	0.945	0.922	0.910	0.938	0.951	0.951	0.929	0.905
		PA_BOOT	0.950	**0.895**	**0.364**	**0.094**	**0.257**	0.934	0.918	**0.357**	**0.095**	**0.268**
		PA_BAYES	0.950	0.905	**0.302**	**0.050**	**0.204**	0.944	0.921	**0.324**	**0.057**	**0.229**
		CPI_BOOT	0.961	0.938	0.997	**0.886**	0.976	0.959	0.940	0.990	0.915	0.978
		UPI_BOOT	0.965	0.943	0.990	0.979	0.993	0.962	0.944	0.987	0.989	0.995
	Power	LMS	0.871	0.919	**0.544**	**0.399**	**0.636**	0.870	0.936	**0.632**	**0.467**	**0.653**
		LMS_BOOT	0.833	0.905	**0.414**	**0.179**	**0.416**	0.838	0.918	**0.483**	**0.259**	**0.467**
		LMS_BAYES	0.853	0.914	**0.579**	**0.538**	**0.694**	0.856	0.919	**0.657**	**0.640**	**0.734**
		PA_BOOT	0.837	0.910	**0.389**	**0.210**	**0.330**	0.832	0.926	**0.418**	**0.218**	**0.343**
		PA_BAYES	0.828	0.917	**0.428**	**0.314**	**0.361**	0.818	0.920	**0.443**	**0.331**	**0.401**
		CPI_BOOT	**0.740**	0.901	**0.017**	**0.082**	**0.033**	**0.716**	0.895	**0.028**	**0.07**	**0.023**
		UPI_BOOT	**0.604**	0.892	**0.007**	**0.017**	**0.006**	**0.627**	0.892	**0.009**	**0.006**	**0.008**
ind	RB	LMS	−0.002	−0.005	0.092	**0.131**	**0.157**	0.009	−0.006	0.064	**0.352**	**0.190**
		LMS_BOOT	−0.002	−0.005	0.092	**0.131**	**0.157**	0.009	−0.006	0.064	**0.352**	**0.190**
		LMS_BAYES	−0.001	−0.007	**0.154**	**0.120**	**0.229**	0.011	−0.006	**0.134**	**0.137**	**0.233**
		PA_BOOT	**−0.269**	−0.074	**−0.620**	**−0.771**	**−0.665**	**−0.262**	−0.078	**−0.612**	**−0.765**	**−0.648**
		PA_BAYES	**−0.270**	−0.076	**−0.622**	**−0.773**	**−0.667**	**−0.267**	−0.084	**−0.617**	**−0.770**	**−0.653**
		CPI_BOOT	−0.005	−0.008	**0.104**	**0.345**	**0.137**	0.009	−0.005	0.081	**0.189**	**0.193**
		UPI_BOOT	−0.001	−0.005	**0.116**	**0.465**	**0.132**	0.014	−0.004	**0.121**	**0.362**	**0.250**
	SE/SD	LMS	1.014	0.969	**0.850**	1.012	0.918	0.961	0.947	0.957	**0.608**	**0.877**
		LMS_BOOT	1.093	1.021	**85.765**	**143.763**	**145.238**	1.027	0.997	**108.643**	**17.925**	**119.731**
		LMS_BAYES	**1.109**	1.029	**11.664**	**35.211**	**16.573**	1.025	1.003	**10.443**	**31.316**	**11.572**
		PA_BOOT	1.008	0.972	0.994	0.917	0.943	0.997	0.959	0.953	**0.855**	0.999
		PA_BAYES	1.022	0.983	0.975	**0.776**	0.915	1.013	0.971	0.931	**0.720**	0.968
		CPI_BOOT	1.085	1.016	**1.530**	0.930	**1.557**	1.037	0.995	**1.690**	**1.172**	0.980
		UPI_BOOT	**1.127**	1.020	**1.389**	**1.116**	**1.309**	1.059	1.000	**1.845**	1.003	**1.391**
	Cov	LMS	0.950	0.939	0.939	0.913	0.941	0.939	0.938	0.948	0.927	0.963
		LMS_BOOT	0.961	0.948	0.973	0.966	0.970	0.954	0.945	0.975	0.972	0.982
		LMS_BAYES	0.959	0.949	0.961	0.978	0.960	0.952	0.946	0.968	0.978	0.972
		PA_BOOT	**0.508**	**0.872**	**0.041**	**0.027**	**0.029**	**0.519**	**0.866**	**0.048**	**0.027**	**0.028**
		PA_BAYES	**0.559**	**0.895**	**0.050**	**0.020**	**0.037**	**0.551**	**0.883**	**0.056**	**0.022**	**0.033**
		CPI_BOOT	0.959	0.947	0.983	**0.889**	0.971	0.954	0.944	0.975	0.915	0.975
		UPI_BOOT	0.962	0.949	0.980	0.976	0.981	0.9600	0.945	0.981	0.974	0.990
	Power	LMS	0.898	0.948	**0.606**	**0.354**	**0.554**	0.909	0.946	**0.617**	**0.369**	**0.584**
		LMS_BOOT	0.874	0.936	**0.367**	**0.192**	**0.348**	0.891	0.936	**0.395**	**0.209**	**0.364**
		LMS_BAYES	0.881	0.932	**0.690**	**0.503**	**0.659**	0.896	0.930	**0.68**	**0.488**	**0.675**
		PA_BOOT	0.977	0.997	0.821	**0.516**	**0.777**	0.978	0.994	0.828	**0.520**	0.805
		PA_BAYES	0.978	0.996	0.839	**0.664**	0.808	0.978	0.994	0.840	**0.657**	0.821
		CPI_BOOT	0.867	0.937	**0.177**	**0.133**	**0.114**	0.893	0.943	**0.237**	**0.124**	**0.154**
		UPI_BOOT	0.852	0.933	**0.135**	**0.064**	**0.088**	0.881	0.942	**0.199**	**0.069**	**0.134**
index	RB	LMS	0.017	−0.003	0.015	0.087	**0.152**	−0.003	−0.003	0.015	0.067	0.095
		LMS_BOOT	0.017	−0.003	0.015	0.087	**0.152**	−0.003	−0.003	0.015	0.067	0.095
		LMS_BAYES	0.030	0.002	0.074	**2.699**	**0.279**	0.007	0.000	0.077	**0.382**	**0.468**
		PA_BOOT	**−0.257**	−0.084	**−0.671**	**−0.831**	**−0.727**	**−0.259**	−0.084	**−0.671**	**−0.826**	**−0.703**
		PA_BAYES	**−0.259**	−0.085	**−0.673**	**−0.833**	**−0.729**	**−0.261**	−0.085	**−0.673**	**−0.828**	**−0.705**
		CPI_BOOT	0.026	−0.004	**0.253**	**0.523**	**0.610**	−0.057	−0.051	−0.092	−0.093	**0.149**
		UPI_BOOT	0.029	−0.001	**0.418**	**−0.269**	**0.920**	−0.064	−0.051	**−0.215**	0.021	0.082
	SE/SD	LMS	0.992	0.911	0.996	**1.183**	0.995	0.944	0.940	1.003	1.015	0.970
		LMS_BOOT	1.053	0.958	**1.140**	**1.236**	**1.200**	1.006	0.991	**1.149**	**1.261**	**1.173**
		LMS_BAYES	1.050	0.963	**2.416**	**1.566**	**2.996**	0.994	0.991	**1.287**	**7.182**	**0.789**
		PA_BOOT	1.021	0.941	1.007	0.925	0.958	0.997	0.971	0.972	0.913	0.981
		PA_BAYES	1.034	0.961	0.954	**0.738**	**0.879**	1.008	0.995	0.927	**0.740**	**0.895**
		CPI_BOOT	1.078	0.979	**2.007**	**1.204**	**1.327**	1.067	1.016	**1.722**	**1.140**	**1.235**
		UPI_BOOT	**1.217**	0.986	**1.592**	**1.443**	**1.231**	**1.151**	1.022	**2.578**	**1.207**	**1.508**
	Cov	LMS	0.946	0.926	0.962	0.949	0.961	0.931	0.939	0.950	0.952	0.950
		LMS_BOOT	0.956	0.937	0.975	0.976	0.980	0.945	0.946	0.973	0.980	0.969
		LMS_BAYES	0.951	0.939	0.956	0.936	0.924	0.941	0.955	0.957	0.937	0.918
		PA_BOOT	**0.817**	0.926	**0.125**	**0.027**	**0.070**	**0.807**	0.917	**0.117**	**0.033**	**0.101**
		PA_BAYES	**0.830**	0.934	**0.109**	**0.017**	**0.058**	**0.820**	0.933	**0.119**	**0.024**	**0.089**
		CPI_BOOT	0.956	0.939	0.997	**0.898**	0.979	0.959	0.943	0.991	0.932	0.978
		UPI_BOOT	0.967	0.945	0.992	0.981	0.993	0.959	0.944	0.989	0.991	0.992
	Power	LMS	0.867	0.916	**0.554**	**0.426**	**0.646**	0.870	0.930	**0.648**	**0.500**	**0.662**
		LMS_BOOT	0.828	0.902	**0.445**	**0.254**	**0.465**	0.837	0.915	**0.538**	**0.336**	**0.519**
		LMS_BAYES	0.853	0.914	**0.579**	**0.538**	**0.694**	0.856	0.919	**0.657**	**0.640**	**0.734**
		PA_BOOT	0.830	0.908	**0.376**	**0.179**	**0.316**	0.826	0.924	**0.397**	**0.187**	**0.328**
		PA_BAYES	0.828	0.917	**0.428**	**0.312**	**0.361**	0.818	0.920	**0.443**	**0.330**	**0.401**
		CPI_BOOT	**0.740**	0.900	**0.020**	**0.080**	**0.033**	**0.715**	0.893	**0.033**	**0.062**	**0.030**
		UPI_BOOT	**0.589**	0.886	**0.010**	**0.015**	**0.006**	**0.620**	0.887	**0.009**	**0.002**	**0.010**

Cor, correlation between X and Z; b_3_, the effect of XZ on Y; ind, the average estimates of the indirect effects at different values of the moderator (a * (b_1_ + b_3_ * Z), whenZ = 0, 1SD, − 1SD); index, the moderated mediation index (a * b_3_).

*Estimator: LMS, Latent moderated structural equation model with robust standard error generated by maximum likelihood estimator; LMS_BOOT, Latent moderated structural equation model with bootstrapped standard error; LMS_BAYES, Latent moderated structural equation model with Bayesian estimation; PA_BOOT, Path analysis with bootstrapping method; PA_BAYES, Path analysis with Bayesian estimation; CPI_BOOT, Constrained product indicator model with bootstrapping method; UPI_BOOT, Unconstrained product indicator model with bootstrapping method*.

*Type: Type 1, Normal distribution; Type 2, Uniform distribution; Type 3, Symmetrical moderated kurtosis distribution; Type 4, Symmetrical high kurtosis distribution; Type 5, Slightly skewed distribution; RB, parameter estimates (b_3_ = 0)/relative bias of the parameter estimates (b_3_ ≠ 0); Cov, 95% coverage. Boldfaced values represent the ones that are out of the acceptable range of the evaluation criteria*.

Similar to study 1, this study focuses on comparing the estimates of the path coefficient (*b*_3_) of the interaction MZ, the moderated mediation index (*a* * *b*_3_), and the average estimates of indirect effects at different values of the moderator (*a* * (*b*_1_ + *b*_3_*Z), *when Z* = 0, 1 *SD*, − 1 *SD*) through the four approaches.

#### Relative Bias

According to Tables 5, 6, results were similar when the correlation between X and Z is 0 or 0.3. Specifically, when the correlation is not zero, the estimates of four approaches were generally better, compared to zero-correlation conditions. Within the conditions of normal distribution and uniform distribution (Types 1 and 2), CPI, UPI and LMS can provide unbiased estimates, LMS had the smallest relative bias when *b*_3_ ≠ 0. When *b*_3_ = 0, the results of LMS and PI were similar. The accuracy of estimates with PA was relatively lower than that of the other three approaches when the parameters of interests were not zero. PA estimation was even less suitable when the data had a symmetrical moderated kurtosis distribution, a symmetrical high kurtosis distribution, or a slightly skewed distribution (Types 3–5). For the Type 3 data, only estimates by LMS with maximum likelihood estimator met the criteria when *b*_3_ ≠ 0.

When cor (X, Z) = 0, for Type 4 data, no approach could provide an acceptable biased estimate of the latent interaction effect when it is not zero. Of the four approaches, LMS's estimates with maximum likelihood estimation had a relatively smaller bias than did the others. When cor (X, Z) = 0.3, CPI could estimate the path coefficient of the interaction effect more accurately than did LMS with a symmetrical high kurtosis distribution (Type 4). For the slightly skewed distributed data (Type 5), all approaches had a relative bias higher than the required standard of 10% when the parameter is not zero. Nevertheless, LMS still presented the smallest relative bias. In general, LMS performed best in terms of the coefficient estimates for all five distributions.

Compared with the maximum likelihood estimation, results did not show any obvious advantages of the Bayesian method in non-normality conditions.

#### Standard Error Ratio

Based on the SE ratio criterion, only the results of LMS with robust standard errors were acceptable under all conditions except for Type 4 data and *b*_3_ = 0.2.

To make a preliminary comparison between the bootstrapped SE and the default SE generated by the ML estimator, we also compared the bootstrapped SE with 100 draws and the default SE in this study. Moreover, to explore whether the SE overestimates were caused by the low number of draws, we also considered bootstrapped SE with 1,000 draws. To save space, we only reported the SE results of this extra investigation. Results showed that PI without the bootstrap method underestimated the standard errors. In comparison, PI with the bootstrap method overestimated the standard errors, thus performing better in coverage rate and worse in power (Table 7). Additionally, the bootstrap method with 1,000 draws still overestimated the SE and could not provide the acceptable SE when data was seriously non-normally distributed. The results were similar to the bootstrap method with 100 draws in most conditions.

**Table 7:** Standard error ratio of *b*_3_ using PI with/without bootstrapped standard errors in study 2 (*b*_3_ = 0.2).

**Cor**	**Type**	**CPI**			**UPI**		
		**Boot 100**	**Boot 1,000**	**Non-boot**	**Boot 100**	**Boot 1,000**	**Non-boot**
0	1	1.077	1.087	0.952	**1.229**	**1.142**	0.920
	2	0.973	1.013	0.926	0.973	1.018	0.922
	3	**1.848**	**1.698**	**0.678**	**1.587**	**1.624**	**0.756**
	4	1.095	**1.108**	**1.279**	**1.496**	**1.241**	**1.222**
	5	**1.341**	**1.698**	**0.735**	**1.392**	**1.376**	1.053
0.3	1	1.064	1.047	0.931	**1.142**	1.082	0.906
	2	1.005	1.003	0.950	1.007	1.005	0.942
	3	**1.758**	**1.771**	**0.787**	**2.493**	**2.076**	**0.791**
	4	0.997	**1.298**	**0.782**	**1.283**	**1.129**	**0.647**
	5	**1.220**	**1.117**	**0.668**	**1.564**	**1.436**	**0.584**

*Cor, correlation between X and Z; CPI, Constrained product indicator model; UPI, Unconstrained product indicator model; Boot 100, Bootstrapped standard error with 100 draws; Boot 1,000, Bootstrapped standard error with 1,000 draws; Non-boot, default standard error used in Mplus*.

*Type: Type 1, Normal distribution; Type 2, Uniform distribution; Type 3, Symmetrical moderated kurtosis distribution; Type 4, Symmetrical high kurtosis distribution; Type 5, Slightly skewed distribution. Boldfaced values represent the ones that are out of the acceptable range of the evaluation criteria*.

#### Coverage Rate

All of the approaches resulted in a coverage rate of approximately 90% in all five conditions except PA. For PA, coverage rates were <90% with non-normally distributed data (Types 2–5) and non-zero parameters.

#### Power

As shown in Table 6, we found that LMS had the highest statistical power for detecting *b*_3_ and the moderated mediation effect (*a* * *b*_3_) among the four approaches under both normal and non-normal conditions. In comparison, PA performed better in detecting indirect effects.

#### Type I Error Rate

The type I error rates of *b*_3_ and *a* * *b*_3_ for the four approaches in the five conditions were between 0 and 0.16. CPI and UPI resulted in a smaller type I error, while PA resulted in a larger type I error especially when the data is symmetrically high kurtosis distributed (see Table 5 for details).

#### Completion Rate

All approaches could reach 100% completion rate for Type 1 and Type 2 data (Table 8). For non-normally distributed data, PA could reach the 100% completion rate for all the conditions except for the Type 4 data when *b*_3_ ≠ 0 and X was not correlated with Z (completion rate = 99.9%). Additionally, LMS also demonstrated a higher completion rate than did PI with non-normally distributed data.

**Table 8:** Completion rate of the four approaches in study 2.

	**Estimator**	**Cor** **=** **0**					**Cor** **=** **0.3**				
***b*_3_**	**Type**	**1**	**2**	**3**	**4**	**5**	**1**	**2**	**3**	**4**	**5**
0	LMS	1	1	0.999	0.95	0.999	1	1	0.998	0.963	0.997
	LMS_BOOT	1	1	0.999	0.95	0.999	1	1	0.998	0.963	0.997
	LMS_BAYES	1	1	0.977	0.621	0.962	1	1	0.997	0.671	0.971
	PA_BOOT	1	1	1	1	1	1	1	1	1	1
	PA_BAYES	1	1	1	1	1	1	1	1	1	1
	CPI_BOOT	1	1	0.993	0.691	0.972	1	1	0.992	0.806	0.988
	UPI_BOOT	1	1	0.605	0.425	0.589	0.999	1	0.674	0.496	0.649
0.2	LMS	1	1	1	0.959	0.998	1	1	0.998	0.976	0.999
	LMS_BOOT	1	1	1	0.959	0.998	1	1	0.998	0.976	0.999
	LMS_BAYES	1	1	0.979	0.652	0.963	1	1	0.994	0.702	0.977
	PA_BOOT	1	1	1	0.999	1	1	1	1	1	1
	PA_BAYES	1	1	1	0.999	1	1	1	1	1	1
	CPI_BOOT	1	1	0.995	0.674	0.97	1	1	0.994	0.791	0.986
	UPI_BOOT	1	1	0.711	0.476	0.67	1	1	0.748	0.539	0.728

*Cor, correlation between X and Z; *b*_3_, the effect of XZ on Y*.

*Estimator: LMS, Latent moderated structural equation model with robust standard error generated by maximum likelihood estimator; LMS_BOOT, Latent moderated structural equation model with bootstrapped standard error; LMS_BAYES, Latent moderated structural equation model with Bayesian estimation; PA_BOOT, Path analysis with bootstrapping method; PA_BAYES, Path analysis with Bayesian estimation; CPI_BOOT, Constrained product indicator model with bootstrapping method; UPI_BOOT, Unconstrained product indicator model with bootstrapping method*.

*Type: Type 1, Normal distribution; Type 2, Uniform distribution; Type 3, Symmetrical moderated kurtosis distribution; Type 4, Symmetrical high kurtosis distribution; Type 5, Slightly skewed distribution*.

## Practical Guidelines of LMS for Continuous Data

The results of the above two simulation studies demonstrated the advantages of LMS over the other three approaches. However, some limitations still hinder the application of LMS. For example, the model fitting cannot be evaluated directly in M*plus*. To promote the use of LMS, we provided the practical guidelines which illustrated how to evaluate the model fitting, and how to adopt the Johnson-Neyman procedure (Johnson and Neyman, 1936) with LMS results. The corresponding M*plus* codes are available in the Supplementary Materials.

Maslowsky et al. (2015) have provided a detailed tutorial for applying the LMS method with the ML estimator. In this section, we simply summarized the guidelines in their paper and illustrated some points that were not covered in it.

### Model Estimation

LMS can be easily implemented in M*plus* using the XWITH command. However, standardized coefficients are not available using M*plus*. Applied researchers can obtain standardized coefficients by standardizing the data.

### Model Fitting

Considering the model fitting in LMS, although it is difficult to evaluate the model fitting directly, there is still an alternative choice based on model comparison. Maslowsky et al. (2015) proposed a two-step method to evaluate model fitting: (1) Conduct the model without interaction term (Model 0) and evaluate the model based on CFI, Tucker-Lewis index (TLI), chi-square value, and so on; (2) add the interaction term (Model 1) and compare the model fitting with Model 0 based on the log-likelihood ratio test. The model fitting of Model 1 is acceptable only when the Model 0 fits data well and the log-likelihood test indicates that Model 1 is better than Model 0. For more details about M*plus* codes of this two-step method, please refer to Maslowsky et al. (2015).

### Bootstrap Confidence Interval

Both the bootstrapped SE or the robust SE can be obtained in LMS, and the corresponding M*plus* codes were available in the Supplementary Materials when the simulation studies were mentioned. However, the current studies showed that LMS with the bootstrap method is time-consuming and overestimates the SE when the reliability is low or when data are non-normally distributed. To obtain robust results, we suggest that applied researchers draw the conclusion based on both the bootstrapped confidence interval and the significance test.

### Johnson-Neyman Technique

The Johnson-Neyman technique is used to obtain the continuously plotted confidence intervals around simple slopes for all values of the moderator. This technique can facilitate the interpretation of results in moderation analysis (Preacher et al., 2007). It can also be implemented in LMS using M*plus*. Its code is available in the Supplementary Materials.

## Discussion

Based on the two simulation studies, we recommend the LMS approach as the optimal choice for the analysis of second-stage moderated mediation models. It can provide relatively unbiased and robust estimates of the latent interaction effect *b*_3_, moderated mediation index *a* * *b*_3_, and indirect effects. This conclusion was proven not only in the condition of low reliability, but also in the case of the violation of the multivariate-normally-distributed assumption.

Of all the simulation studies conducted, the low reliability study produced results with higher relative bias and a larger SE ratio, plus a lower statistical power and completion rate. Although PA is a simple approach for researchers to grasp and implement, measurement errors cause issues for its application. When the reliabilities were approximately 0.8 (the accepted reliability level for a social science scale), PA with ML estimator showed an 8.45% underestimation of the interaction effect when *b*_3_ ≠ 0. The bias of this estimate rose to 56.68% when reliability decreased to 0.6. It seems that PA suffers from an increasing bias of its estimate with the decreasing reliability of the data. This issue has also been identified by previous research (Cheung and Lau, 2017). Although PA performed worst in the estimates, a PA approach (Ng and Chan, 2020) based on the factor scores was shown to efficiently estimate latent moderation models. Ng and Chan (2020) also found that this factor score method provided unbiased estimates of the moderation effect when the composite reliability was just acceptable. Further studies are suggested to investigate the performance of this factor score approach in moderated mediation models, and to test whether PA's good performance in detecting mediation effects remains.

The reduction in reliability also had a negative impact on the PI analysis of the interaction effect. It is reasonable to expect that the process of PI, which requires the multiplication of indicators, would widen the effect of measurement errors. This was evident in our investigation; decreasing the reliability of the data resulted in increasing bias of the respective estimates. By contrast, LMS simply conducts the interaction with the original data in the latent-variable framework. It not only considers the effect of measurement errors, but also limits the influence of the errors on the estimation. Therefore, the estimates of the LMS approach were still acceptable under conditions of varying reliabilities (Cham et al., 2012).

Study 2 compared the four approaches with four different types of non-normally distributed data: uniform distribution, symmetrical moderated kurtosis distribution, symmetrical high kurtosis distribution, and moderated skewed distribution. We took this approach because the dependent variable, X, may be correlated with the moderator, Z, in real data analysis. We also considered the possibility of these variables being correlated simultaneously with the condition of non-normally distributed indicators. The estimates were similar to the conditions when X was not correlated with Z. The results indicated that the LMS approach was the optimal choice for all of these situations. For the normally distributed and uniformly distributed data, the PI and LMS approaches both demonstrated only a minor level of bias in their estimates (Wall and Amemiya, 2001; Marsh et al., 2004; Cheung and Lau, 2017). When the data follow the symmetrical moderated kurtosis distribution, LMS provides the estimates with the least biases (Klein and Moosbrugger, 2000; Cheung and Lau, 2017). For the symmetrical high kurtosis distributed and slightly skewed distributed data, estimates could not meet the criteria with any approach. However, compared with the other three approaches, LMS was the most suitable in estimating moderation effects. Although it also failed to fall in the ideal interval, it still maintained an acceptable balance between the accuracy and standard errors for the estimation. A possible explanation for this may be that LMS conducts the interaction directly, without product indicators, whereas these indicators are required and need to follow the multivariate-normally distributed assumption for other methods. When the data are non-normally distributed, the violation of the assumption would create biased estimates for standard errors, significance tests, and intervals of the parameters under the PI analysis (Klein and Moosbrugger, 2000).

The violation of the assumption of normally distributed data would more severely influence the estimation of indirect effects than would the interaction effects among all the approaches. All four approaches provided unacceptably biased estimates of indirect effects. These results were consistent with previous studies (Cham et al., 2012; Aytürk et al., 2019). A recent study showed that the application of instrumental variables in latent moderation models can reduce the influence of non-normality (Brandt et al., 2020). However, since this method cannot be easily applied in M*plus*, we suggest that applied researchers use this method with R software when facing data that seriously violates the normality assumption. Moreover, the performance of the instrumental variables has not been investigated thoroughly in moderated mediation models, and therefore more simulation studies are needed in the future.

Compared with the ML estimation, the advantages of Bayesian analysis in small sample size conditions were demonstrated in study 1. However, while providing the best estimates among all the approaches, Bayesian estimation also tends to overestimate the standard errors of indirect effects, thus reducing the power. Moreover, the Bayesian method did not perform better than the ML estimator with non-normally distributed data in study 2. This research just made a preliminary exploration of the different performances between the traditional ML method and the Bayesian method in moderated mediation models. More studies are needed to compare these two methods in other settings, and to investigate whether the disadvantages of Bayesian LMS in estimating standard errors can be circumvented by informative priors. Moreover, since the posterior predictive *p*-values are not available in latent moderated mediation models, the model fitting of Bayesian analysis suggest these models also need to be further explored.

Considering the standard error estimates, LMS with robust standard errors performed better than bootstrapped standard errors in estimating indirect effects, especially with low reliability and small sample size. The overestimates of standard errors made the bootstrap method fail to provide acceptable power in these conditions. PI with bootstrapped standard errors also demonstrated the overestimates similar to LMS with the bootstrap method with non-normally distributed data. However, the number of bootstrap draws used in this study is small, so future studies should investigate whether the problem of overestimating can be circumvented by increasing the number of bootstrap draws.

Model fitting is also a problem existing in all the latent approaches applied for moderated mediation models. While LMS with M*plus* cannot provide indices for evaluation of the model fitting directly, there are still alternative methods. In addition to the two-step method proposed by Maslowsky et al. (2015), Gerhard et al. (2017) also proposed a new index for detecting omitted non-linear terms (quadratic and interaction terms) in latent moderation analysis. Moreover, although PI can provide the commonly used indices in SEM, Mooijaart and Satorra (2009) also showed that the chi-square test is insensitive when detecting the interaction effect using the PI method.

For this research, we still have much more to do with the integrated model. Although this study has already discussed the moderated mediation model under the conditions of low reliability, non-normal distribution, and the correlation between independent and moderator variables, it could not cover all situations for actual studies. Future studies may focus more on other types of the integrated model, such as the first-stage moderated mediation model, to investigate whether the performances of the four estimation approaches are similar. Since these are all complex models, the appropriate approaches for different conditions may vary. The conditions in this study were also limited; conclusions could be enriched with datasets with categorical and/or missing data, more complex manipulation of the moderated mediation index, and the consideration of correlation with measurement errors, and so on. Future studies are also suggested to include more indicators per factor to compare the performance of matching and parceling strategies in moderated mediation models. Moreover, the advantages of LMS in handling the missing values compared to PI were also demonstrated in the previous study (Cham et al., 2017). Since Bayesian estimation has advantages in dealing with missing values (Pan et al., 2017), future research should further investigate the performance of these methods in such conditions.

In sum, although PA is the most widely applied approach for the moderated mediation model in practical studies, it is strongly biased by the ignorance of measurement errors. CPI and UPI could both provide acceptable estimates when the multivariate normal distribution assumption holds. It is strongly suggested to apply LMS in practical research, as it could provide precise estimates for parameters, and powerful conclusions under conditions such as low reliability, non-normal distributions of data, and correlations between variables. However, when facing seriously non-normally distributed data, no approach in this paper can provide accurate estimates, and thus no method is appropriate for application in these conditions. The practical guidelines also illustrated the implementation of LMS in detail, especially on how to obtain the bootstrapped confidence interval, how to implement the Johnson-Neyman technique, and how to evaluate model fitting. This research could help to broaden the application of LMS in psychology and social science research, and to provide researchers with an emerging tool to generalize their conclusions.

## Data Availability Statement

Simulation datasets were analyzed in this study. All the simulation materials including data generation, analysis, and evaluation measures can be found in the Supplementary Materials.

## Author Contributions

QF, QS, LZ, and JP designed and executed the study, conducted the data analyses, and wrote the manuscript. LZ and JP made substantial contributions in conducting additional simulation studies and revising the manuscript critically. QS, LZ, SZ, and JP critically reviewed and revised the manuscript. QF, QS, and LZ are the co-first authors and contributed equally to this work. All authors approved the final version of the manuscript for submission.

## Conflict of Interest

The authors declare that the research was conducted in the absence of any commercial or financial relationships that could be construed as a potential conflict of interest.

## Footnotes

^1^The model constraints used in PI are not available with Bayesian estimation in M*plus*.

^2^Because it is very time-consuming to use the bootstrap method in latent moderated structural equation modeling, bootstrapped standard errors are computed just using 100 draws. Under conditions of low reliability and 1000 samples with 100 bootstrap draws, it took more than a month to complete one simulation condition on a tower server with Intel Xeon E5-2620 CPU and 32-Gbit memory.
