# Supplementary material for: Integration of Moderation and Mediation in a Latent Variable Framework: A Comparison of Estimation Approaches for the Second-Stage Moderated Mediation Model
Source: Front Psychol. 2020 Sep 10;11:2167. doi: 10.3389/fpsyg.2020.02167 (PMC7511593; doi:10.3389/fpsyg.2020.02167)
Supplement: Supplementary file 1 [file Data_Sheet_1.ZIP › r_code_sub/Readme.pdf]

Readme

All the datasets in the simulation studies were generated in R (R Devel- opment Core Team, 2013) and all the models were conducted using Mplus 8.4 (Muthén & Muthén, 1998-2017). This file briefly introduces the R codes for generate the datasets, conduct analysis and extract results in simulation studies.

Data Generation

"data_gen" folder

- "def_con.r": define the sample size, number of observed variables, number of factors, etc
- “true.r”: population values of parameters (i.e., variances, loadings, coefficients)
- "data_gen.r": generate datasets

Modeling

"mplus_gen" folder: generate mplus file for running different models

- "write_mplus_cpi.r": write the mplus code for constrained product indicator (CPI) analysis
- "write_mplus_upi.r": write the mplus code for unconstrained product indicator (UPI) analysis
- "write_mplus_lms.r": write the mplus code for latent moderated structural modeling (LMS)
- "write_mplus_reg.r": write the mplus code for path analysis (PA)

Results

- "results_extract.r": extract results from mplus output: the relative bias, se/sd, power, mean square error, coverage rate of estimates, completion rate, etc.

Reference

- Muthén, L. K., & Muthén, B. O. (1998-2017). *Mplus user's guide. Eighth Edition.* Los Angeles, CA: Muthén & Muthén.
- R Core Team. (2013). *R: A language and environment for statistical computing*. Vienna, Austria: R Foundation for Statistical Computing.
